# An orthogonalized PYR1-based CID module with reprogrammable ligand-binding specificity

**DOI:** 10.1038/s41589-023-01447-7

**Published:** 2023-10-23

**Authors:** Sang-Youl Park, Jingde Qiu, Shuang Wei, Francis C. Peterson, Jesús Beltrán, Angélica V. Medina-Cucurella, Aditya S. Vaidya, Zenan Xing, Brian F. Volkman, Dmitri A. Nusinow, Timothy A. Whitehead, Ian Wheeldon, Sean R. Cutler

**Affiliations:** 1 Department of Botany and Plant Sciences, University of California, Riverside, Riverside, CA USA; 2 Institute for Integrative Genome Biology, University of California, Riverside, Riverside, CA USA; 3 Department of Biochemistry, University of California, Riverside, Riverside, CA USA; 4 Department of Biochemistry, Medical College of Wisconsin, Milwaukee, WI USA; 5 Department of Chemical Engineering and Materials Science, Michigan State University, East Lansing, MI USA; 6 Donald Danforth Plant Science Center, St. Louis, MO USA; 7 Department of Chemical and Biological Engineering, University of Colorado, Boulder, CO USA; 8 Department of Chemical and Environmental Engineering, University of California, Riverside, Riverside, CA USA; 9 Center for Industrial Biotechnology, University of California, Riverside, Riverside, CA USA; 10 Present Address: Department of Plant and Soil Sciences, Delaware Biotechnology Institute, University of Delaware, Newark, DE USA

**Keywords:** Synthetic biology, X-ray crystallography

## Abstract

Plants sense abscisic acid (ABA) using chemical-induced dimerization (CID) modules, including the receptor PYR1 and HAB1, a phosphatase inhibited by ligand-activated PYR1. This system is unique because of the relative ease with which ligand recognition can be reprogrammed. To expand the PYR1 system, we designed an orthogonal ‘*’ module, which harbors a dimer interface salt bridge; X-ray crystallographic, biochemical and in vivo analyses confirm its orthogonality. We used this module to create PYR1*^MANDI^/HAB1* and PYR1*^AZIN^/HAB1*, which possess nanomolar sensitivities to their activating ligands mandipropamid and azinphos-ethyl. Experiments in *Arabidopsis thaliana* and *Saccharomyces cerevisiae* demonstrate the sensitive detection of banned organophosphate contaminants using living biosensors and the construction of multi-input/output genetic circuits. Our new modules enable ligand-programmable multi-channel CID systems for plant and eukaryotic synthetic biology that can empower new plant-based and microbe-based sensing modalities.

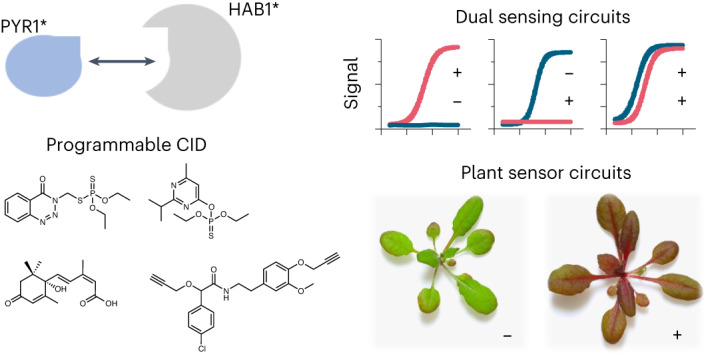

## Abstract (web summary)

Park et al. redesigned the abscisic acid-induced dimerization module to respond to diverse ligands and function orthogonally to the natural modules, enabling synthetic biological circuit design in plants and yeast.

## Main

Systems for regulating biological responses with small molecules have broad utility. Starting with the *Escherichia coli* lac operon in the 1970s, many synthetic ligand-regulated circuits have been built by rewiring natural systems for new functions^1^. In principle, numerous biological parts can be reprogrammed to construct chemically controlled functions, including ligand-induced transcription factors (for example, *lacI)*, cell surface receptors and their downstream signaling components (for example, GPCRs and DREADDs and analog-sensitive kinases) and chemical-induced dimerization (CID) systems (for example, rapamycin/FKBP/FRB), among many possibilities^2–4^. Of these, CID is particularly attractive because it provides modular parts that can be used to engineer chemically regulated transcription, enzyme activity, protein localization, stability and other processes^2,5–8^.

The first described CID systems involve microbe-derived dimerizing ligands, such as rapamycin, that direct protein neo-associations that rewire eukaryotic cell signaling^5^. Plant biologists subsequently discovered that many phytohormones regulate signaling through CID mechanisms. For example, auxin acts as a molecular glue to stabilize a complex between the ubiquitin ligase SCF^TIR1^ and downstream AUX/IAA transcriptional co-repressors, which leads to their ubiquitylation and degradation^9^. This mechanism is also exploited by the phytohormone jasmonic acid and is analogous to the thalidomide-mediated degradation of transcription factors that underpin the anti-cancer activities of immunomodulatory ligands^10–13^. In the auxin and jasmonic acid systems, ligand recognition is shared between protein-binding partners. In contrast, the receptors for ABA, gibberellic acids and strigolactones are allosteric switches that first bind their ligands within solvent-excluded pockets and then form complexes with effector proteins^14–16^. Several plant hormone sensors (for example, ABA, auxin and gibberellic acid) have been co-opted to engineer chemically regulated processes^2^. Of these, the ABA perception system is the most extensively characterized at the structural and biochemical levels^17^.

ABA is a stress hormone perceived by the soluble receptor PYR1 (encoded by *Pyrabactin resistance 1*) and its related *PYR1-*like and *Regulatory* *Component* *of* *ABA* *Receptor* proteins (*PYL/RCAR*, *PYLs* for simplicity)^18^. ABA binds to PYL receptors and stabilizes activated conformers that bind and inhibit type 2C protein phosphatases (PP2Cs), which prevents the PP2Cs from dephosphorylating and inactivating their downstream stress-activated SnRK2 kinase targets^14,19–22^. The ABA sensor module is particularly powerful for biosensor design^23–25^. We previously developed multiple agrochemical-regulated CID modules, including PYR1^MANDI^, designed to enable agrochemical control of plant ABA signaling^25^. More recently, we used computationally guided mutagenesis to develop PYR1 sensors for 21 structurally diverse cannabinoids and organophosphates, and others reprogrammed a different PYL to sense 12 herbicides^23,24^. Thus, PYLs have malleable, reprogrammable ligand-binding pockets. In addition, dozens of studies have used the ABA sensor to construct ABA-regulated processes in mammals, yeasts and bacteria^2,26–37^. Thus, this scaffold is unique because it merges an easily reconfigurable binding pocket with plug-and-play deployment as a chemical-induced dimerizer.

The relative ease of developing new PYR1-based biosensors stems from two interconnected biochemical properties: affinity amplification and dimerization. PYR1’s phosphatase partner acts analogously to a co-receptor, boosting apparent ligand-binding affinity up to ~100-fold^20,38^. This makes ligand recognition relatively easy to reprogram, as a few mutations are sufficient for new binding specificities^23,25,39^. Because ligand recognition can be altered without disrupting the dimer interface, affinity amplification is ligand independent and requires only a ligand to stabilize PYR1’s activated conformer. In this study, we exploited this modularity to redesign the PYR1/HAB1 dimer interface to create a new orthogonal ‘*’ CID system. We show here that we can reprogram the orthogonal module’s ligand recognition and use it with the wild-type (WT) module to construct two-channel control of *Saccharomyces* *cerevisiae* transcriptional outputs. In addition, we developed Arabidopsis plants that visually report the presence of nM levels of azinphos-ethyl and diazinon, two banned organophosphate agrochemicals. Thus, our new modules will facilitate the development of plants and microbes that sense and respond to user-specified chemical signals and expand the growing set of biological parts available for plant synthetic biology^40–42^.

## Results

### Design of an orthogonal ABA-regulated PYR1/HAB1 sensor

**Fig. 1:**
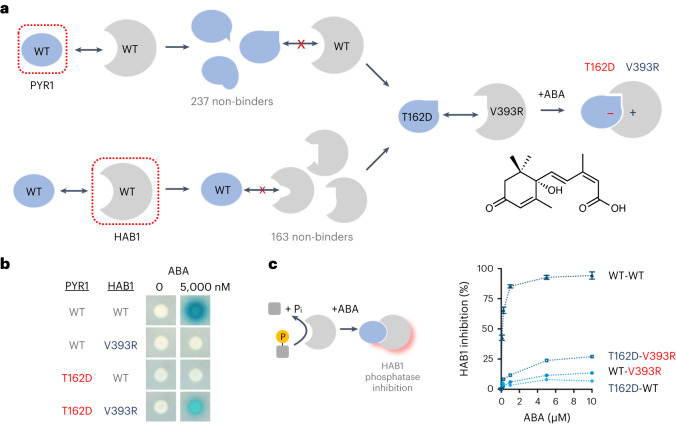
Construction of an orthogonal PYR1/HAB1 dimerization module using an allele-specific suppression strategy. **a**, Design pipeline for identification of ‘orthogonalizing’ mutations. The WT ABA sensor contains an ABA receptor, PYR1, that binds to the PP2C HAB1 in response to ABA to form a stable complex. A two-step approach was taken to identify mutations that program an orthogonal PYR1/HAB1 interaction. Site saturation mutagenesis of both proteins was first used to systematically identify mutations at the PYR1/HAB1 binding interface that disrupt ABA-mediated dimerization using established Y2H assays; this identified 237 and 163 non-binding mutants in PYR1 and HAB1, respectively (Supplementary Tables 1 and 2). The collection of non-functional mutant alleles was co-transformed into a Y2H reverse two-hybrid strain, and mutually suppressive allele combinations were identified using a growth-based selection strategy. The best suppressor pair identified was PYR1^T162D^/HAB1^V393R^ (Supplementary Table 3 shows all combinations identified). **b**, Orthogonal interactions between the WT and mutant modules. PYR1^T162D^ does not bind to HAB1, nor does HAB1^V393R^ bind to PYR1, as measured using Y2H assays of the ABA-stimulated interaction (ABA 5,000 nM; mock, DMSO carrier solvent control). Colony overlay assays with an X-gal substrate indicate Y2H circuit activation by β-galactosidase induction. **c**, HAB1 phosphatase inhibition assay for combinations of WT and mutant alleles using a 4-methylumbeliferyl phosphate (4-MUP) substrate. Data points indicate the mean of three technical replicates; error bars indicate the s.d. and are normalized to the HAB1 activity in the presence of the receptor tested but without ligand. The data demonstrate that PYR1^T162D^ does not cross-activate WT HAB1, as expected for an orthogonalizing mutation. Source data

We leveraged Y2H-based selection systems to create an ABA-regulated PYR1/HAB1 CID module that functions orthogonally to the WT sensor. We defined a large set of dimer interface mutant alleles that disrupt ABA-stimulated interactions to do this. Then, we used functional selections to identify mutually suppressive allele combinations that restore ABA-induced dimerization (Fig. 1a). To systematically define these mutations, we screened a previously constructed 399-member, indexed library of PYR1 mutants that harbor saturating mutations in 21 residues at the HAB1-binding interface^43^. We similarly constructed a collection of 266 saturating HAB1 mutations targeting 14 PYR1-binding residues. Each mutant was tested for ABA-stimulated dimerization with its WT binding partner using Y2H assays, which identified 236 and 163 mutations that impair ABA-induced dimerization in PYR1 and HAB1, respectively (Supplementary Tables 1 and 2). These collections of ‘dead’ alleles were co-transformed into an *S. cerevisiae* reverse two-hybrid strain^44^, and growth-based selections were used to select mutant combinations that restore ABA-induced dimerization. This identified nine pairs of mutually suppressive allele combinations, the best of which was PYR1^T162D^/HAB1^V393R^ (Fig. 1b and Supplementary Table 3). T162D/V393R pack against one another in the WT module, and our structural studies (described below) show that the mutant pair introduces a salt bridge that stabilizes PYR1^T162D^/HAB1^V393R^ binding while presumably destabilizing both PYR1^T162D^/HAB1 and PYR1/HAB1^V393R^ interactions (see below).

In land plants, where we originally discovered the ABA-sensing PYR1/HAB1 CID module, activation of endogenous signaling requires receptor-mediated phosphatase inhibition, which leads to activation of downstream stress-activated SnRK2 kinases^19^. To investigate PYR1^T162D^/HAB1^V393R^ orthogonality using a biochemical assay, we conducted in vitro phosphatase assays using recombinant proteins and observed that PYR1^T162D^ does not strongly regulate HAB1’s activity, consistent with its behavior in Y2H assays (Fig. 1c). In addition, we observed that PYR1^T162D^ does not regulate HAB1^V393R^ activity, despite its ability to bind HAB1^V393R^ in Y2H assays (Fig. 1c); this implies that the ABA-activated PYR1^T162D^ conformer is unable to block substrate access to HAB1^V393R^’s catalytic pocket. PYR1^T162D^’s inability to regulate HAB1’s catalytic activity should limit interactions between native ABA and engineered orthogonal responses in plant-based CID applications, a point that we return to later in the context of designing plant genetic circuits. Thus, our mutagenesis and functional selection scheme yielded a mutation pair that orthogonalized the PYR1/HAB1 CID module.

### Orthogonalized PYR1/HAB1 has a malleable ligand-binding pocket

**Fig. 2:**
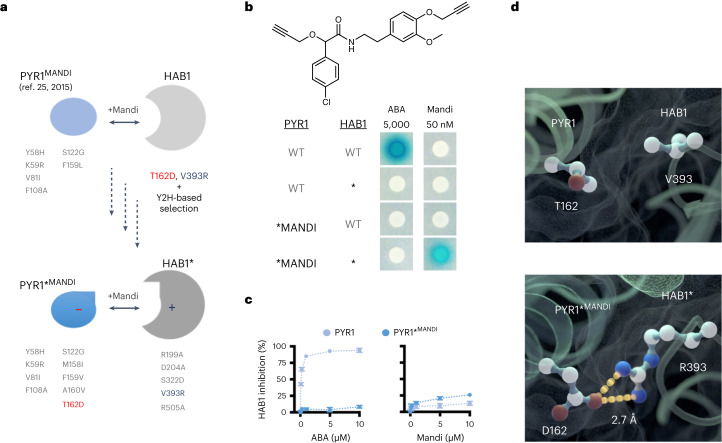
Construction of an orthogonal mandi-regulated PYR1*^MANDI^/HAB1* dimerization module. **a**, Design pipeline for the development of PYR1*^MANDI^/HAB1*. **b**, Y2H assays of WT and orthogonal module components. **c**, PYR1*^MANDI^ does not regulate WT HAB1 PP2C activity as measured using a 4-MUP substrate; data points indicate the mean of three technical replicates; and error bars indicate the s.d. The WT PYR1/HAB1 ABA response control in the left graph is the same as that used in Fig. 1c, as the datasets were acquired at the same time. **d**, The crystal structure of a PYR1*^MANDI^/mandi/HAB1* ternary complex demonstrates that the T162D/V393R orthogonalizing mutant pair installs a salt bridge. Coordinates for a PYR1-ABA-HAB1 complex (PDB 3QN1) were used to illustrate the WT interface. The images were rendered in Cinema3D using meshes exported from Pymol. Source data

**Extended Data Fig. 1:**
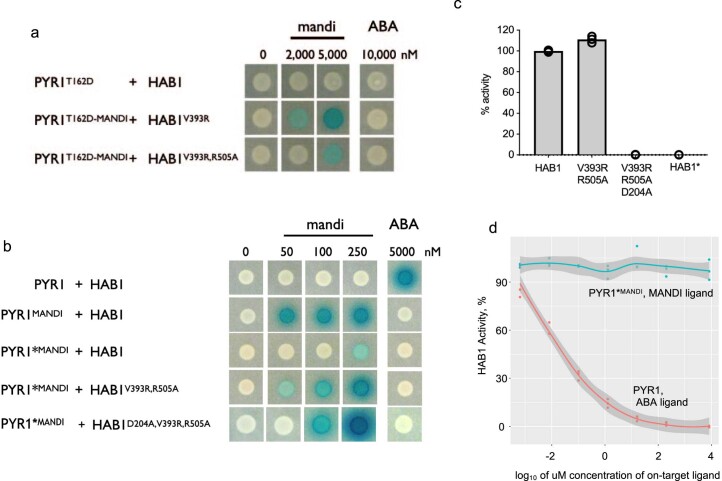
Characterization of interactions between module components identified along the directed evolutionary trajectory to the PYR1*^MANDI^/HAB1* CID module. (**a**, **b**) Y2H interaction assays using the mutant CID components shown. These assays use derivatives of pBD-PYR1 and pACT-HAB1 and are assayed in the S. cerevisiae Y2H reporter strain Y190. Colony overlay assays with an X-gal substrate were used to visualize β-galactosidase activity. The yeast strains were tested on different concentrations of the compounds listed (DMSO carrier solvent in the mock 0 µM control wells). (**c**) Recombinant GST-HAB1 and the mutants shown were produced in *E. coli*, and phosphatase activity was measured using a spectrophotometric assay with a 4-MUP substrate, as described in the methods; activity is reported as %GST-HAB1 control. Bars show the mean of triplicate assays, and error bars are the standard deviation. (**d**) Recombinant GST-HAB1, 6x-His-PYR1, and 6X-His-PYR1*^MANDI^ were produced in E. coli, and phosphatase activity was measured using a phosphopeptide substrate and malachite green; these data confirm that PYR1*^MANDI^ does not inhibit HAB1 PP2C activity. The x-axis shows log10 of the ABA or mandi concentrations tested (µM). Activity is plotted relative to the mock control, which lacks test ligands but contains a receptor and phosphatase; 95% confidence intervals are shown. Source data

To determine if the orthogonalized module’s ligand recognition could be reprogrammed, we ‘grafted’ the T162D/V393R mutations onto the previously engineered receptor PYR1^MANDI^ and HAB1 (Fig. 2a). We tested PYR1^MANDI,T162D^ for its interactions with HAB1^V393R^ and HAB1^V393R,R505A^ (R505A blocks interactions with PP2C-regulated kinases^45^ and was introduced to reduce ABA cross-talk in plant applications). The mutant receptor responded to mandi in Y2H assays but with ~100-fold reduced sensitivity compared to the PYR1^MANDI^/HAB1 interaction (Extended Data Fig. 1). We next used mutagenesis and selection to improve the mandi sensitivity of PYR1^MANDI,T162D^ (Fig. 2a). Pilot screens that targeted mutagenesis to both interface and ligand-contacting residues (see Methods for details) indicated that sensitivity could be increased by C-terminal mutations of M158 and A160, which are near the orthogonalizing T162D and the mandi-sensitizing F159L mutations, both located in PYR1’s C-terminal α-helix. Based on this, we hypothesized that T162D/F159L mutations reduced mandi sensitivity by altering the local structure of the C-terminal α-helix of PYR1, which participates in ligand-induced conformational changes and is positioned to contact the bulky orthogonalizing HAB1^V393R^ mutation^46^. We, therefore, mutagenized M158, F159 and A160 in the C-terminal α-helix and identified mutations in these residues that increase module sensitivity using Y2H-based selections, yielding the nonuple mutant PYR1*^MANDI^ (PYR1^Y58H;K59R;V81I;F108A; S122G;M158I;F159V;A160V;T162D^). To further limit any potential effects of HAB1’s phosphatase activity on cellular processes, we incorporated a catalytic D204A mutation^26^, creating HAB1^V393R,R505A,D204A^, which eliminated phosphatase activity but also reduced mandi sensitivity (Extended Data Fig. 1a). Additional mutagenesis of HAB1^V393R,R505A,D204A^ followed by positive selections led to a catalytically inactive, pentuple mutant that we call HAB1* (HAB1^R199A;D204A;S322D;V393R;R505A^). The final PYR1*^MANDI^/HAB1* CID module responds to low nM mandi concentrations in Y2H assays, selectively binds to HAB1* and does not strongly regulate WT HAB1’s phosphatase activity, which together indicate sensitive and orthogonal function of the final PYR1*^MANDI^/HAB1* CID module (Fig. 2b,c). The complete amino acid sequences of PYR1*^MANDI^ and HAB1* are provided in Supplementary Table 4.

**Extended Data Fig. 2:**
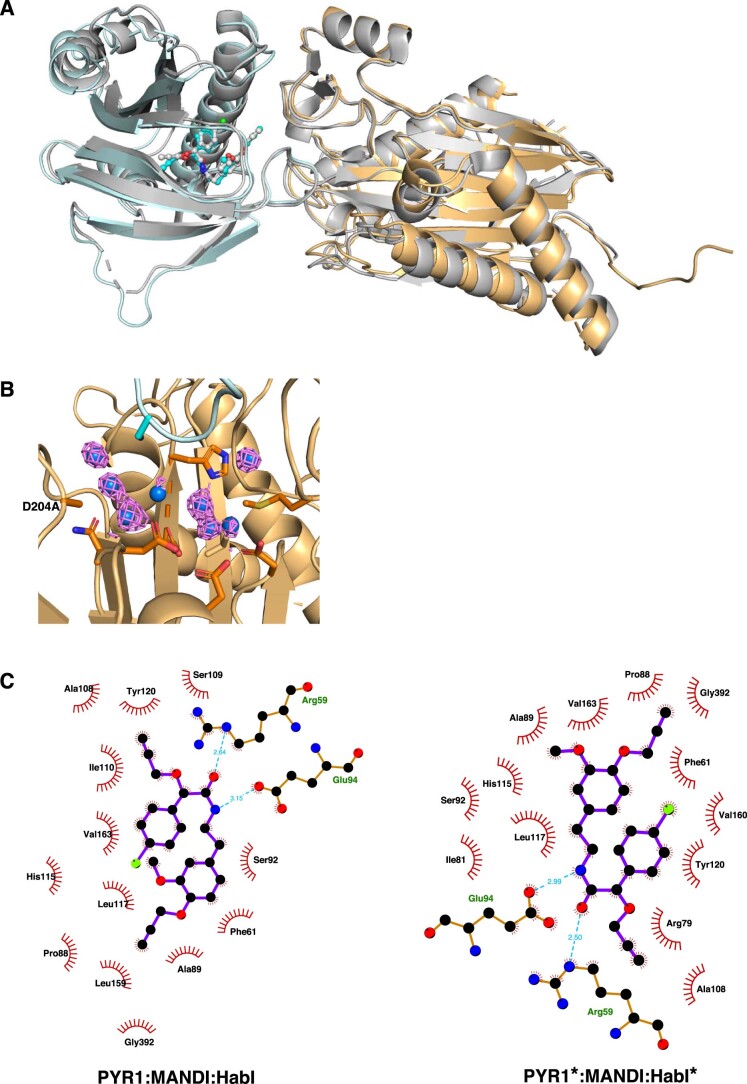
HAB1* X-ray crystal structure analysis. (**A**) Alignment of the PYR1:mandipropamid:HABI ternary complex (gray), PDB ID 4WVO, with the PYR1*^MANDI^:mandipropamid:HABI* ternary complex (PYR1*^MANDI^ – cyan, HABI* – orange). The two ternary complexes have an RMSD value of 1.43 Å when 457 of the 470 CA atoms are used in the superimposition. (**B**) The catalytic pocket of HABI* (orange) lacks sufficient electron density to support the presence of metal ions required for catalytic activity. The available density is only sufficient to support a network of water molecules (blue spheres). The tip of the PYR1*^MANDI^ gate is shown in cyan. (**C**) Interaction of PYRI (left panel) or PYRI*^MANDI^ (right panel; referred to in this panel as PYR1*) with mandipropamid in their respective ternary complexes.

The extensive mutagenesis required to engineer high-sensitivity mandi responsiveness prompted us to obtain an X-ray crystal structure of a PYR1*^MANDI^/mandi/HAB1* ternary complex, which we solved at 2.4-Å resolution. As expected, the ligand–receptor contacts mirror those of the original PYR1^MANDI^; the module is nearly superimposable with WT modules (CA RMSD of 1.7 Å); and the electron density map confirms the absence of metal ions in HAB1’s catalytic site (Extended Data Fig. 2). The structure reveals the consequences of the orthogonalizing T162D/V393R mutations, which form a salt bridge positioned 2.7 Å from one another (Fig. 2d). Thus, our mutational pipeline created an orthogonal signaling module by introducing a salt bridge in the PYR1*^MANDI^/HAB1* binding interface with minimal changes to the module’s global structure.

**Fig. 3:**
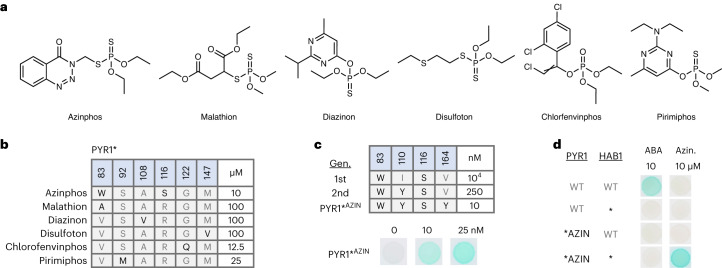
PYR1* has a malleable ligand-binding pocket. **a**, Structures of ligands screened. To establish if the new ligand-responsive receptors could be isolated using the orthogonal module, PYR1*^MANDI^ was subjected to saturating mutagenesis using NNK oligonucleotides. The resultant library was screened using a reverse two-hybrid strain against a panel of 10 organophosphate ligands at 100 μM, and hits were retested and sequenced. **b**, The table shows the changes to PYR1*^MANDI^’s ligand-binding pocket (and neighboring residues) for the organophosphate receptors identified (Y2H and sequence data are provided in Extended Data Fig. 3); the right-hand side of the table shows the minimal concentration that yielded a positive signal in β-gal-based Y2H assays. **c**, An azinphos-ethyl-responsive mutant was subjected to two rounds of affinity optimization using Y2H-based selections. The mutations identified at each round of mutagenesis and selection are shown; the minimal ligand concentration yielding a positive signal in β-gal-based Y2H assays is shown at the right. The bottom panel shows β-gal-based Y2H assays conducted on PYR1*^AZIN^. **d**, The PYR1*^AZIN^/HAB1* module functions orthogonally to the WT ABA response module. Shown are pair-wise tests of the WT or * components with one another tested for activation by ABA or azinphos-ethyl (10 µM). Gen., generation.

**Extended Data Fig. 3:**
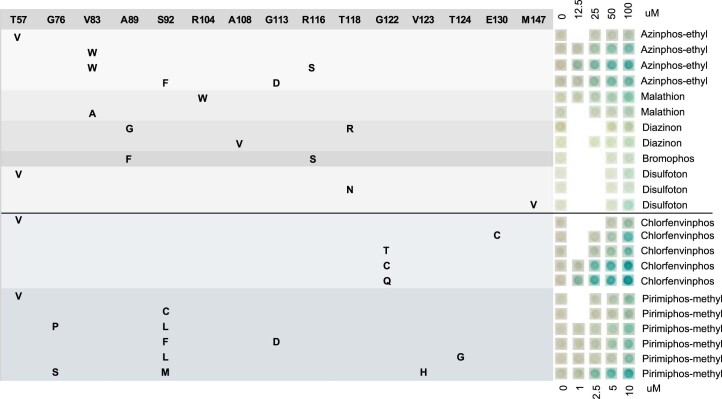
Screens of an NNK-mutagenized PYR1*^MANDI^ library yield receptors for diverse organophosphate ligands. Characterization of organophosphate responsive sensors isolated by screening a library of NNK-mutagenized PYR1*^MANDI^ variants. The mutations in each sensor isolated are shown in the table, and the parental PYR1*^MANDI^ sequences are shown at the top. Y2H assays using different ligand concentrations are shown on the right. The mutants characterized were isolated in growth-based Y2H screens and showed reproducible ligand-stimulated growth; however, a small number of the clones show marginal β-galactosidase activity in the assays. This table includes these because the initial growth on 100 µM ligand was reproducible.

**Extended Data Fig. 4:**
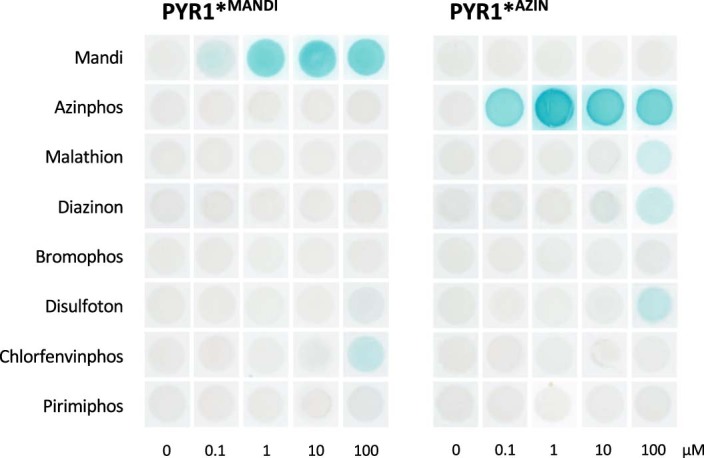
PYR1*^MANDI^ and PYR1*^AZIN^ cross-reactivity with organophosphates. PYR1*^MANDI^ and PYR1*^AZIN^ were tested for specificity using Y2H experiments with different concentrations of organophosphates (0, 0.1, 1, 10, and 100 µM) and stained with X-gal by chloroform overlay.

We next set out to establish if PYR1*^MANDI^’s binding pocket could be engineered to bind new ligands similarly to the WT receptor^23^. To do this, we used NNK mutagenesis to construct a PYR1*^MANDI^ single-site saturation mutagenesis library targeting the entire coding sequence, allowing both ligand-contacting and proximal residues that modulate ligand binding to be identified. The resultant library was screened against a panel of 10 organophosphate ligands, targets of interest for environmental sensing, to yield receptors responsive to six of the 10 organophosphate ligands tested (Fig. 3a,b and Extended Data Fig. 3). An azinphos-ethyl receptor was selected for affinity maturation and subjected to two rounds of recombination-based mutagenesis and selection to yield a pentuple mutant (PYR1*^MANDI, N15E, V83W, I110Y, V164Y, V190Y^; PYR1*^AZIN^) that responds to nM concentrations of azinphos-ethyl in Y2H assays (Fig. 3c). PYR1*^AZIN^ additionally retains tight orthogonality and does not interact with WT HAB1 (Fig. 3d). In addition, we tested PYR1*^AZIN^ and PYR1*^MANDI^ for activation by seven organophosphate ligands using Y2H assays and observed minimal cross-activation at concentrations up to 100 µM, indicating high ligand selectivity (Extended Data Fig. 4). The complete amino acid sequences of PYR1*^AZIN^, PYR1*^MANDI^ and HAB1* are provided in Supplementary Table 4. Thus, our new orthogonal scaffold can be engineered to respond to multiple ligands, similarly to the WT module.

### PYR1* and PYR1 enable orthogonal genetic circuits

**Fig. 4:**
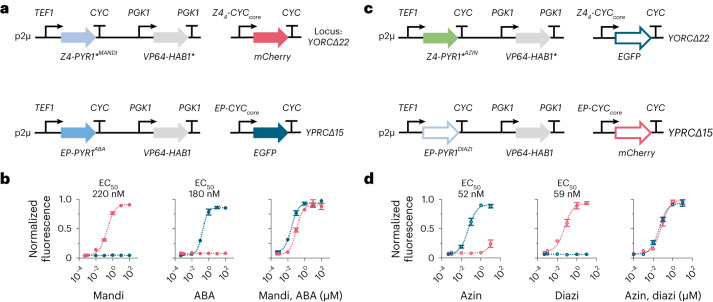
PYR1/PYR1*-enabled multi-input/output circuits in *S. cerevisiae*. **a**, Genetic design of tandem ABA and mandi-regulated transcriptional circuits. The gene, promoter and terminator for each expression cassette are listed. PYR1* was fused to the synthetic zinc finger DNA-binding protein Z4, whereas PYR1 was fused to the EP DNA-binding domain. VP64 transcriptional activation domains were fused to HAB1 and HAB1* to enable transcription of the reporter genes, mCherry in the PYR1* circuit and EGFP in the PYR1 circuit. Reporter genes were integrated into the genome as indicated, whereas the circuit components were expressed from a 2µ plasmid with *TEF1p* expressing PYR1*/*PYR1* and *PGK1p* for HAB1*/*HAB1*. **b**, Ligand-mediated induction of the reporter genes was measured 12 h after ligand addition. Fluorescence was normalized to the maximum signal of each reporter (mCherry: 37,100 RFU; EGFP: 67,900 RFU). Red data points represent the response of the PYR1*^MANDI^ circuit; blue points represent the response of the PYR1 circuit. **c**, Genetic design of tandem azinphos-ethyl and diazinon-regulated circuits. DNA-binding domains were fused to PYR1 and PYR1* as described in **a**, but, in this case, EGFP was used as a reporter with PYR1*, and mCherry was used with the PYR1 module. **d**, Ligand-mediated induction of *EGFP* and *mCherry* reporter genes for the tandem PYR1*^AZIN^ and PYR1^DIAZI^ circuits. Fluorescence was normalized to the maximum signal of each reporter (EGFP: 17,700 RFU; mCherry: 14,100 RFU). Red data points represent the response of PYR1*^AZIN^, whereas blue data points represent the response of PYR1^DIAZI^. All data points in **b** and **d** represent the mean of three biological replicates using independent transformants and were collected by flow cytometry (see Extended Data Fig. 3 for gating details). Error bars represent the s.d. RFU, relative fluorescence units. Source data

To establish if the orthogonal * response modules could be used for multi-input/output transcriptional responses in vivo, we designed and tested WT and * module-controlled circuits in both *S. cerevisiae* and *Arabidopsis* *thaliana*. In yeast, we enabled dual circuit control by using the Z4 zinc finger domain and the macrolide repressor protein from *E. coli* (here referred to as EP) to drive the expression of two separate reporter genes^47,48^. Circuits were constructed by fusing EP to PYR1 and Z4 to PYR1*^MANDI^ or PYR1*^AZIN^ and the transcriptional activation domain VP64 to HAB1 and HAB1* (Fig. 4a). Using the WT and PYR1*^MANDI^ modules, we created a set of circuits that respond to ABA and mandi with nM sensitivity and negligible cross-talk (Fig. 4b). Replacing PYR1 with PYR1^DIAZI^, a diazinon-responsive receptor previously described^23^, and PYR1*^MANDI^ with PYR1*^AZIN^ produced a single yeast strain that responded to one or both of these organophosphate-based pesticides with low nM half-maximal effective concentration (EC_50_) values and large dynamic range (Fig. 4c,d). Given the large number of organophosphates that the PYR1 and PYR1* scaffolds can be made to sense (7–10 have been shown for each receptor; see Extended Data Fig. 3) (ref. ^23^), this opens the possibility of creating multi-sensor strains that can report on the presence and concentration of these environmental contaminants. Collectively, these data demonstrate that newly evolved PYR1-based and PYR1*-based switches can be combined to regulate distinct outputs with minimal cross-talk. This feature should broaden the complexity of CID applications accessible with this scaffold.

**Extended Data Fig. 5:**
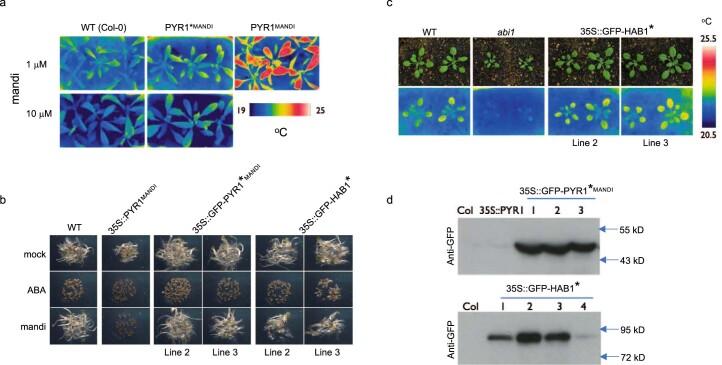
Characterization of PYR1*^MANDI^ and HAB1* overexpression effects. (**a**) Col-0 wild type and two 35S::GFP-PYR1*^MANDI^ strains were treated with 1 µM mandipropamid, thermographed after 24 hours, treated seven days later with 10 µM mandi, and again imaged 24 hours later. A previously constructed 35 S::PYR1^MANDI^ transgenic line was included as a positive control (1 µM treatment only). These images show that mandipropamid treatments do not cross active endogenous ABA signaling in the 35S::GFP-PYR1*^MANDI^ transgenic lines; the positive control 35S::PYR1^MANDI^ strain shows a strong thermal response to ABA, as previously described; the experiment was conducted one time. (**b**) Seed germination responses of wild type, 35S::PYR1^MANDI^, 35S::GFP-PYR1*^MANDI^, and 35S::GFP-HAB1* transgenic strains to ABA (0.5 µM), mandipropamid (0.5 µM), or mock treatments. Mandi inhibits germination of 35S::PYR1^MANDI^ seeds due to activation of ABA signaling but does not in 35S::GFP-HAB1* strains. (**c**) Overexpression of wild-type HAB1 causes ABA insensitivity, which can be observed indirectly as reduced leaf temperature caused by elevated transpiration. Two 35S::GFP-HAB1* transgenic lines were grown alongside wild-type Columbia and abi1 (Col-0) as a positive control (it possesses growth defects and reduced leaf temperature due to aba insensitivity). The GFP-HAB1* plants show leaf temperatures comparable to wild-type plants, indicating that the GFP-HAB1* protein does not strongly impair plant guard cell ABA responses. (**d**) GFP-PYR1*^MANDI^ and GFP-HAB1* protein levels were established by western blotting using an anti-GFP antibody; as described above, the three lines had similar protein expression levels. The experiment was conducted once, and the entire scanned gel is presented in the supplemental information. Source data

The design of ligand-regulated circuits for plant synthetic biology is limited by the relatively small number of parts available for building genetic circuits^49,50^. Our * module may address this; however, its utility requires tight insulation of * components from the endogenous ABA signaling machinery. Because ABA signaling has many physiological effects, such as inhibiting seed germination and triggering guard cell closure, cross-activation by the * module could cause numerous unwanted ABA-related phenotypes. Our module incorporates several mutations to limit cross-talk. First, the T162D/V393R orthogonalizing mutations reduce */WT interactions. Second, catalytic inactivation of HAB1* by the D204A mutation prevents it from dephosphorylating SnRK2 kinases and blocking ABA signaling. Third, HAB1* contains two mutations (R505A and V393R) that disrupt its ability to bind SnRK2s^45^. Lastly, PYR1*^MANDI^ is not activated by ABA due to several mutations in its binding pocket (Fig. 2b,c). We constructed and analyzed 35S::GFP-PYR1*^MANDI^ and 35S::GFP-HAB1* transgenic plants to investigate potential cross-talk. Seed germination and leaf temperatures (which increase due to guard cell closure) of the 35S::GFP-PYR1*^MANDI^ plants are similar to WT after ABA or mandi treatments, unlike a previously constructed *35S::PYR1*^*MANDI*^ positive control^25^ (Extended Data Fig. 5a,b). Similarly, the leaf temperatures and morphologies of 35S::GFP-HAB1* lines are similar to WT, unlike an ABA-insensitive mutant (*abi1-1*) positive control (Extended Data Fig. 5c) or the insensitivity phenotypes previously documented when overexpressing HAB1 and related PP2Cs^51,52^. Collectively, these observations indicate negligible ABA pathway cross-talk by the * module components.

**Fig. 5:**
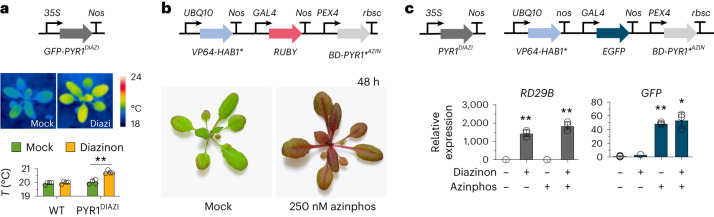
PYR1/PYR1*-enabled multi-input/output circuits in *A. thaliana*. **a**, Regulation of leaf temperature by PYR1^DIAZI^. The gene, promoter and terminator used in the circuits are shown. PYR1^DIAZI^ controls native ABA signaling by ligand-induced binding to cellular PP2Cs, as previously demonstrated for PYR1^MANDI^ (ref. ^25^). A single-insert homozygous 35S::GFP-PYR1^DIAZI^ line (line 1) was treated with diazinon (500 nM) or mock (DMSO carrier solvent + surfactant), and leaf temperatures were imaged by thermography 24 h after treatment. Increased leaf temperatures occur due to ABA-induced guard cell closure, which reduces transpirational cooling; quantification of thermographs is shown; data are presented as mean values ± s.d. (**P = 0.001; two-tailed Student’s *t*-test comparing mock diazinon-treated plant leaf temperatures; *n* = 4 biological replicates). **b**, A PYR1*^AZIN^-based circuit drives RUBY gene expression in Arabidopsis in response to azinphos-ethyl. Shown are data from a single-insert homozygous transgenic line (line 3) with a GAL4-based PYR1*^AZIN^/HAB1* circuit driving expression of the betalain pigment marker RUBY (see diagram above images) treated with 250 nM azinphos-ethyl and imaged 48 h after treatment. The data shown are a subset of a larger experiment involving two independent transgenic lines shown in Extended Data Fig. 6. **c**, Two-circuit control gene expression in Arabidopsis. A 35S::PYR1^DIAZI^, PYR1*^AZIN^/HAB1* transgenic line was constructed by transforming PYR1*^AZIN^/HAB1* plants with a 35S::PYR1^DIAZI^ construct and selecting for homozygous double transgenic lines in the F_3_ generation. Five-day-old seedlings were transferred to MS plates containing mock, diazinon (500 nM), azinphos-ethyl (100 nM) or both compounds together (500 nM diazinon, 100 nM azinphos-ethyl); RNA was extracted after 24 h; and marker gene expression was measured by qRT–PCR, normalizing relative to AtPEX4. Plots show expression of treatments relative to the mock control, presented as mean values ± s.d. RD29B is a well-characterized ABA-responsive gene, and its induction indicates PYR1^DIAZI^ activation by diazinon. Experiments were conducted using transgenic line L1 in biological triplicate. Comparisons to the mock control were made by one-way ANOVA with Dunnett’s correction for multiple comparisons. RD29: **P = 0.0092, 0.0098; GFP: *P = 0.0254, **P = 0.0023. Source data

**Extended Data Fig. 6:**
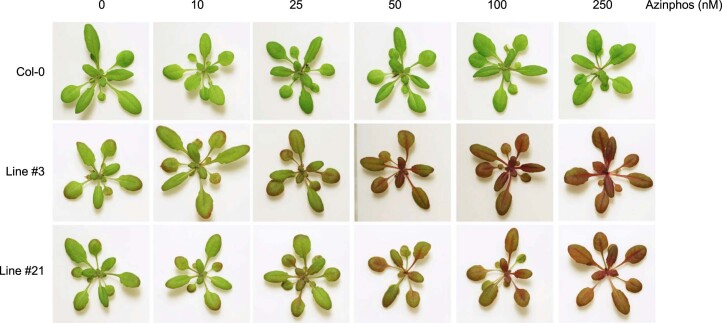
A PYR1*^AZIN^-based circuit drives RUBY gene expression in Arabidopsis in response to azinphos-ethyl. Shown are images collected using two independent, single-insert homozygous transgenic lines with a GAL4-based PYR1*^AZIN^/HAB1* circuit driving expression of the betalain pigment marker RUBY. Foliar applications of 0, 10, 25, 50, 100, and 250 nM azinphos-ethyl to eight plants of Col-0, line #3, and line #21 were imaged 48 hours post-treatment. All solutions contain 0.05% DMSO (carrier solvent) and 0.02% Silwet (a surfactant added with agrochemicals to improve chemical uptake into inner tissues). The images show one representative seedling per treatment.

**Extended Data Fig. 7:**
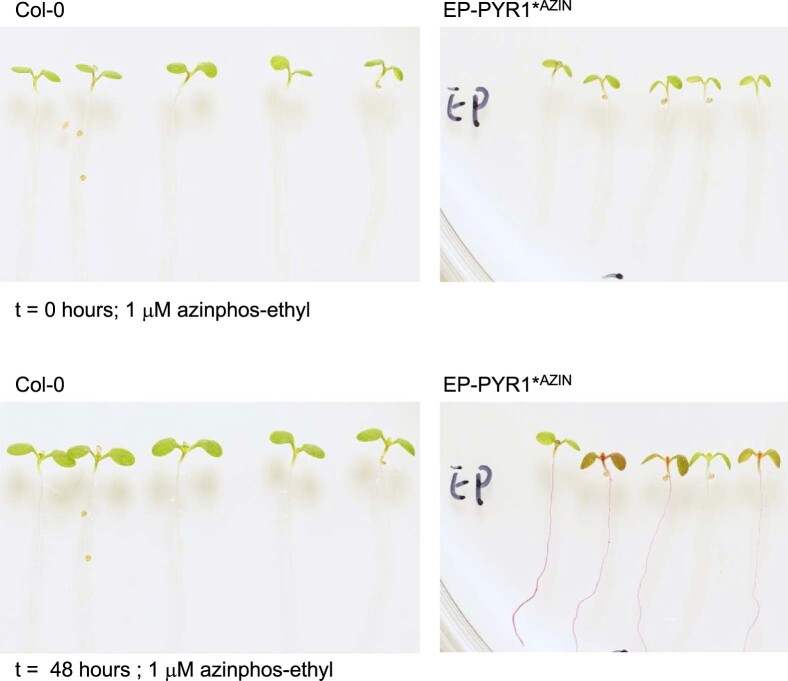
Columbia-0 plants transformed with the EPDBD-PYR1*^AZIN^/ VP64-HAB1* module can control gene expression. Five primary transgenic RFP^+^ seedlings were identified after germination, transferred onto media containing azinphos-ethyl (1 µM), and imaged immediately after transfer (t = 0) and 48 hours later. Four of the five transgenic seedlings display azinphos-ethyl-induced RUBY pigmentation.

To further investigate the suitability of the * module for plant synthetic biology, we used our engineered PYR1 and PYR1*/HAB1* variants to construct transgenic plants where diazinon and azinphos-ethyl regulated distinct outputs. We constructed two independent *35S::GFP-PYR1*^*DIAZI*^ single-insert transgenic lines and observed diazinon-induced increases in leaf temperature (Fig. 5a) and growth inhibition (Supplementary Fig. 1), as expected based on prior characterization of PYR1^MANDI^ (ref. ^25^). To validate the ‘*’ module, we constructed multiple transgenic lines where *GAL4*_*DBD*_PYR1*^AZIN^/VP64-HAB1*-based constructs drive the expression of the synthetic betalain pigment biosynthetic gene *RUBY*^53^ or *EGFP*; these lines showed marked changes in pigmentation or GFP expression after exposure to low nM azinphos-ethyl concentrations by foliar application to plants grown in soil or seedlings in Petri dishes (Fig. 5b, Extended Data Fig. 6 and Supplementary Fig. 2). We also constructed an E-protein-controlled version of this system (*EP*_*DBD*_PYR1*^AZIN^/VP64-HAB1*). Similarly, we observed *RUBY* induction in four of five primary transgenic plants after exposure to 1 µM azinphos-ethyl (Extended Data Fig. 7). Thus, the PYR1^*AZIN^/HAB1* module can be combined with the GAL4 or E-protein systems to construct azinphos-ethyl reporter plants.

**Extended Data Fig. 8:**
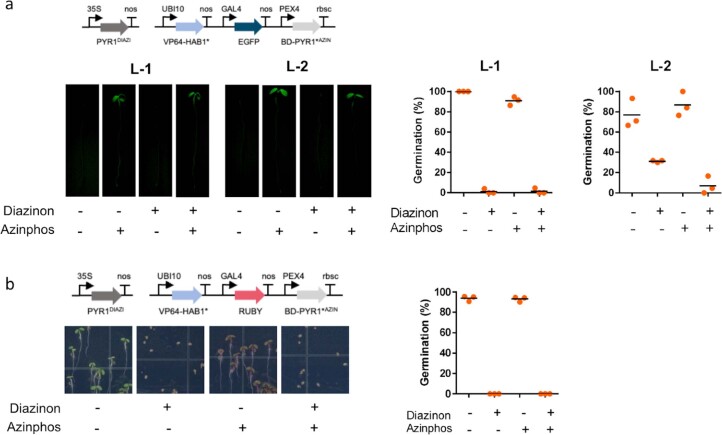
Generating and characterizing multiple transgenic plant lines for dual circuit plants. (**a**) Ligand-mediated circuit activation in dual sensor Arabidopsis plants. PYR1^DIAZI^ was incorporated into plants harboring the PYR1*^AZIN^-GFP inducible circuit in response to azinphos-ethyl by floral transformation, obtaining transgenic lines harboring both circuits (L1 and L2). (left) Five day-old seedlings were treated with either mock, diazinon, azinphos-ethyl, and combined diazinon and azinphos-ethyl, each at (500 nM). Seedlings were imaged 48 hours post-treatment (images all corrected for brightness at 40%). (right) Double senor lines were tested for diazinon-mediated activation of the ABA pathway by germination assays in media containing ligands. Germination was recorded after three days. (**b**) Ligand-mediated circuit activation in dual sensor Arabidopsis plants. PYR1^DIAZI^ was incorporated into plants harboring the PYR1*^AZIN^-RUBY inducible by crossing and then identifying the homozygous double circuit line in the F_2_. (left) Seeds were germinated on ½ MS media containing either mock, diazinon, azinphos-ethyl, and combined diazinon and azinphos-ethyl, each at (500 nM) for five days. (right) Double senor lines were tested for diazinon-mediated activation of the ABA pathway by germination assays in media containing ligands. Germination was recorded after three days. Data points represent the % germination from approximately 100 seeds. Source data

**Extended Data Fig. 9:**
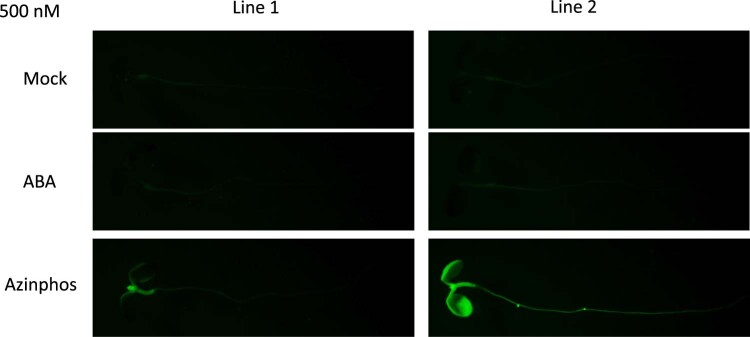
ABA does not cross-activate a PYR1*^AZIN^/HAB1*-controlled circuit in double-circuit transgenic lines. ABA cross-activation in the double circuit transgenic lines (L1 and L2) was characterized by germinating seedlings on 1/2X MS agar Petri plates, growing them under continuous illumination in a growth cabinet, transferring the seedlings 5-days post-germination to Petri plates containing either mock (carrier solvent only), ABA, or azinphos-ethyl (500 nM), and imaged 48 hours post-application under identical camera settings on a fluorescence dissecting microscope equipped with NightSea illumination and filter sets.

To investigate multi-input/output genetic circuits in plants and potential cross-talk between the multiple circuits, we constructed two double transgenic lines homozygous for both *35S::PYR1*^*DIAZI*^ and *GAL4*_*DBD-*_PYR1*^AZIN^/VP64-HAB1*, which drives a *GAL*_*UAS*_*::EGFP* reporter (Fig. 5c). qRT–PCR analyses show that diazinon treatment selectively activates the expression of *RD29B* (an ABA-responsive marker gene) with negligible cross-activation of the PYR1*^AZIN^-controlled GFP reporter. Similarly, azinphos-ethyl treatments selectively triggered the expression of GFP mRNA without activating ABA marker gene expression (Fig. 5c and Extended Data Fig. 8). We also observed similar selectivity in a dual transgenic line with *35S::PYR1*^*DIAZI*^/PYR1*^AZIN^-controlled RUBY reporter (Extended Data Fig. 8). We did not observe ABA-mediated activation of the *AZIN circuit (Extended Data Fig. 9), indicating effective insulation of the module from the ABA pathway. This result is consistent with our observations made analyzing transgenic plants overexpressing the PYR1*^MANDI^ and HAB1* module pieces (shown in Extended Data Fig. 5). Thus, the WT and * parts function individually and when combined.

## Discussion

CID systems are powerful because they enable the robust design of chemically regulated processes. The first CID systems were built from immunosuppressant building blocks (rapamycin, FKBP and FRB)^2,5^; subsequently, new dimerization systems emerged from plant hormone sensors after the discoveries that auxin, gibberellic acid (GA), ABA and others act through induced dimerization mechanisms^2,54^. The ABA system is attractive because its ligand-binding specificity can be reprogrammed with relatively few mutations. Unlike molecular-glue-style dimerizers, where ligand recognition is shared between two proteins, the ABA system uses a single receptor, simplifying redesign efforts^23–25^. Collectively, these features enable the design of new receptors and CID systems built around user-specified ligands.

We set out to develop an orthogonal ABA signaling module to allow multi-channel CID. To do this, we employed a genetic strategy. We first identified collections of PYR1/HAB1 interface mutations that debilitate their interactions and then used a positive selection to identify mutually suppressive combinations. Subsequent modifications to allow high-affinity sensing with a catalytically inactive HAB1 variant led to a final optimized ‘*’ module. The new module retains the WT’s programmability, allowing us to develop multiple agrochemical/pesticide-regulated variants using a simple directed evolution pipeline. Thus, we developed two new agrochemical-regulated CID modules, PYR1*^MANDI^ and PYR1*^AZIN^, and an engineering pipeline for developing new ones.

Our design of the PYR1*/HAB1* module incorporated features to enable CID in plants. Neither the ABA, the GA, nor the auxin CID systems are suitable for plant applications because of the bioactivity of their ligands. We previously developed PYR1^MANDI^ (ref. ^25^), which allows a non-native ligand to activate signaling; however, PYR1^MANDI^ has a WT interface that binds to endogenous plant phosphatases and, therefore, cannot be used as a generic synthetic biology controller (except outside of plants^37^). Our ‘*’ modules incorporate interface mutations in PYR1 and HAB1 that block binding to WT partners, a catalytic mutation in HAB1 that prevents dephosphorylation of endogenous ABA-related targets, and SnRK2-binding site mutants in HAB1 that restrict ABA-related effects of the module. We think that these design efforts were successful because overexpression of the engineered components in Arabidopsis did not affect multiple ABA-related phenotypes (for example, seed germination or transpiration/leaf temperature).

These advances open the door to many exciting applications in plant synthetic biology. For example, we leveraged the programmability and orthogonality of the PYR1 systems to create indicator plants for the banned pesticide azinphos. We first engineered a ‘*’ sensor for azinphos and then used it to create transgenic plants harboring a genetic circuit where PYR1*^AZIN^/HAB1* drives expression of the beet pigment betalain; these transgenic plants become visibly pigmented in response to low nM concentrations of foliar-applied pesticide. PYR1*^AZIN^/HAB1* also enabled sensitive azinphos detection in *S. cerevisiae* (EC_50_ 52 nM), and can be combined with other PYR1 sensors for multi-channel chemical responses. Thus, the PYR1*/HAB1* module provides a scaffold for creating eukaryotic genetic circuits controlled by user-defined ligands and designing multi-input/output CID systems that function seamlessly across biological kingdoms.

## Methods

### Site saturation mutagenesis

We screened a previously constructed collection of saturating mutations in PYR1’s HAB1-binding interface to identify mutations that disrupt ABA-induced binding^43^, using the pBD-PYR1/pACT-HAB1^19^ system in *S. cerevisiae* strain Y190 (ref. ^55^). Test strains were spotted onto agar plates containing SD -Leu,-Trp (SD-LT) media supplemented with 10 μM ABA and stained by chloroform lysis 2 d after growth at 30 °C (see ref. ^56^ for a detailed protocol); ‘dead’ mutants were defined by lack of visible staining in comparison to a WT pBD-PYR1 control (Supplementary Table 1). A HAB1 site saturation mutagenesis library targeting interface residues was made using QuickChange mutagenesis with ‘NNK’ mutagenesis primers and a pACT-HAB1 template^19^. This generated an indexed collection of sequence-verified saturating mutants that we transformed into Y190(pBD-PYR1) and then tested for ABA responsiveness as described above (Supplementary Table 2). The primers used are provided in Supplementary Table 6, plasmids in Supplementary Table 7 and strains in Supplementary Table 8.

### Recombinant protein production

PYR1 and PYR1^T162D^ were expressed from pET28 as 6×-His-tagged protein; HAB1 and HAB1^V393R^ were cloned with a primer set Hab1 cd5 EcoRI and Hab1 cd3 XhoI into pGEX-4T-1 and expressed as a GST fusion protein. Recombinant PYR1*^MANDI^ was cloned into pET28 to generate a 6×-His-tagged receptor; HAB1* was cloned into pGEX-4T-1 to create a GST fusion protein. Proteins were expressed in BL21[DE3]pLysS *E. coli* host cells at 18 °C overnight and purified from sonicated lysates using Ni-NTA agarose (Qiagen) or Pierce glutathione agarose (Thermo Fisher Scientific). His-tagged receptor proteins were resuspended in buffer A (50 mM NaH_2_PO_4_, 300 mM NaCl, pH 8.0) supplemented with 10 mM imidazole and a cleared lysate prepared after sonication and centrifugation. The lysate was applied to the Ni-NTA column and washed with buffer A supplemented with 30 mM imidazole and eluted with buffer A supplemented with 100 mM imidazole. GST-tagged Hab1 proteins were expressed with growth media supplemented with 1 mM MnCl_2_; cleared lysates were prepared in TBS supplemented with 10 mM MnCl_2_, which was included through all purification steps. The lysate was applied to a glutathione agarose column, washed with TBS and eluted with TBS containing 20 mM reduced glutathione. The eluted proteins were dialyzed against TBS at 4 °C. The protein concentration was determined by absorbance at A_280_ using a NanoDrop (Thermo Fisher Scientific).

### PP2C assays

PP2C assays were conducted using 100 nM 6×-His–PYR1 or 6×-His–PYR1^T162D^, 50 nM GST-HAB1 or 50 nM GST-HAB1^V393R^, 100 mM Tris-HCl (pH 7.9), 100 mM NaCl, 1 mM MnCl_2_, 1% β-mercaptoethanol and 0.3% BSA. Reactions were mixed with ABA, mandipropamid or mock DMSO carrier solvent and equilibrated for 30 min, after which 4-methylumbelliferyl phosphate was added (1 mM final concentration). The plates were read using a Victor 2 plate reader (PerkinElmer) (355 nm excitation, 460 nm emission), and assays were run in triplicate. PP2C activity values are reported as percent mock control values, calculated using the carrier solvent (1% DMSO) and the specific receptor/PP2C assayed but no ligand. We note that the recombinant GST-HAB1^V393R^ has ~50% activity per milligram of protein compared to GST-HAB1. Additional assays of PYR1*^MANDI^ activity were conducted using a phosphopeptide-based substrate and malachite green detection, which was conducted using 6×-His-tagged PYR1 or PYR1*^MANDI^, prepared as described above, and 6×-His-ΔN-HAB1 (HAB1 lacking residues 1–179, expressed from pETM-11) using a commercial assay kit (Promega). Reactions contained 125 nM ΔN-HAB1, 250 nM receptor and 50 µM substrate in the manufacturer’s assay buffer. Reactions were incubated for 20 min at room temperature and stopped by adding 50 μl of manufacturer’s molybdate dye/additive mixture and were read spectrophotometrically at 600 nm 1 h later.

### Detection of GFP-PYR1*^MANDI^ and GFP-HAB1* proteins in transgenic Arabidopsis

The proteins expressed as GFP fusion proteins were extracted in TBS from 4-day-old seedlings, and 30 µg of total proteins was separated by SDS-PAGE. After transfer of proteins onto nylon membrane, proteins were detected using an anti-GFP monoclonal antibody (JL-8, Clontech) and a horseradish peroxidase (HRP)-linked anti-mouse IgG secondary antibody from sheep (GE Healthcare). Both antibodies were used at 1/1,000 dilution. Signal was detected using enhanced chemiluminescence (PerkinElmer).

### Yeast transformation and growth

Transformed yeast cells were acquired by first inoculating 5 ml of liquid media and incubating in a shaker incubator overnight at 30 °C. Cells from the overnight culture were then used to inoculate a 50-ml culture with an initial cell density of 5 × 10^6^ cells per milliliter. This culture was grown at 30 °C with 200-r.p.m. shaking until ~2 × 10^7^ cells per milliliter were produced, approximately 3–5 h. Cells were harvested by centrifugation at 4,000 r.p.m. for 5 min, washed with 25 ml of sterile, deionized (DI) water, resuspended in 1 ml of 100 mM LiAc and transferred to a 1.5-ml tube, producing a cell suspension with approximately 10^9^ cells per milliliter. The cell suspension was briefly mixed by vortexing, and 100 µl was transferred into a clean 1.5-ml tube for each transformation. Cells were pelleted at 13,000 r.p.m. for 15 s, and the supernatant was removed. A transformation mixture of 240 µl of PEG (50% w/v), 36 µl of 1.0 M LiAc, 50 µl of salmon sperm DNA (2.0 mg ml^−1^), plasmid and sterile water was added to the cell pellet and mixed by vortexing for 1 min (total volume of 360 µl). The mixed transformation solution was then heat shocked at 42 °C for 40 min. After heat shock, cells were recovered by centrifugation at 8,000 r.p.m. for 30 s. Cells resuspended in 400 µl of sterile water were plated on appropriate selection media—in this case, SD-U media. Plates were incubated at 30 °C until mature colonies were formed.

### Identification of orthogonalizing mutations

To identify mutually suppressing mutant pairs (that is, pairs of allele-specific suppressors) from the collection of non-functional HABl and PYR1 interface mutants, we prepared a pool of plasmid DNAs for the collection of mutant HAB1s and transformed the pool into the *S. cerevisiae* strain Y190, yielding ~3,200 colonies. This collection of ~3,200 subsequently transformed with pooled pBD-PYRl-dead mutant plasmids to generate a library of ~400,000 yeast colonies containing random mutant–mutant combinations. The pooled cells were plated onto selective SD-LTH media supplemented with 10 mM 3-aminotriazole and 10 μΜ ABA to isolate clones harboring mutant combinations that interacted strongly enough to support growth. This identified seven pairs of ABA-dependent interacting mutants (Supplementary Table 3), the strongest of which was the PYR1^T162D^/HAB1^V393R^ pair, which showed an interaction strength that was ~4-fold lower than WT PYRl/HAB l control in β-galactosidase staining assays (Fig. 1). This orthogonal PYR1^T162D^/HAB1^V393R^ mutant pair was selected for subsequent engineering.

### Construction of PYR1*^MANDI^

To select for mutations that improve the sensitivity of PYR1^T162D-MANDI^, we used NNK mutagenesis targeting P55, H60, F61, I62, K63, V83, S85, G86, L87, P88, A89, S92, E94, I110, H115, R116, L117, Y120, E141, P148, G150, N151, D154 and D155. Preliminary screens indicated that M158I and A160C increase mandi sensitivity. Given their proximity to F159L present in PYR1^MANDI^, we subsequently targeted mutagenesis to M158, F159 and A160 using a degenerate NNK-codon primer and QuickChange mutagenesis using a pBD-PYR1^T162D-MANDI^ template (which harbors the following mutation in PYR1: Y58H, K59R, V81I, F108A, S122G, F159L, T162D). A library of ~12,000 clones was generated and transformed into *S. cerevisiae* strain MaV99 harboring pACT-HAB1^V393R,R505A^. Approximately 20,000 transformants were obtained, pooled and then plated onto SD-LT agar media supplemented with 0.1% 5-fluoroorotic acid (FOA) to select against mutants that interact in a ligand-independent fashion. MaV99 is a previously described reverse two-hybrid strain^44^. The surviving cells were collected and plated onto SD-LTU plates supplemented with 50 nM mandi to identify mandi-responsive receptors. Colonies displaying uracil-independent growth were isolated and retested to isolate PYR1*^MANDI^ (Y58H, K59R, V81I, F108A, S122G, M158I, F159V, A160V, T162D). The complete amino acid sequence for this receptor is provided in Supplementary Table 4.

### Construction of HAB1*

To limit the potential interference of HAB1 with endogenous signaling in target organisms, we introduced a catalytic site mutation by QuickChange site-directed mutagenesis to generate pACT-HAB1^V393R,D204A,R505A^. This mutant had reduced sensitivity when tested against PYR1*^MANDI^ and was, therefore, ‘tuned’ for improved sensitivity by mutagenesis and functional selections. To do this, we conducted 28 individual QuickChange NNK mutageneses of the pACT-HAB1^V393R,D204A,R505A^ targeting interface residues (R199, S200, E201, E203, D243, G244, H245, G246, G247, S322, E323, T324, D346, K365, K381, I383, Q384, W385, Q386, R389, F391, G392, Y404, S431, G433, D436, D492 and N493). The resultant 28 libraries were pooled, transformed into *S. cerevisiae* strain MaV99(pBD-PYR1*^MANDI^) and then plated onto selective agar media SD-LTU + 500 nM mandi. Three mutations in two separate residues (S322D, S322E and R199A) were identified that enhance mandi responsiveness. S322D and R199A were combined using the Lightning Multi-site Mutagenesis (Agilent) method to yield the final, high-sensitivity pentuple mutant HAB1* (R199A, S322D, V393R, D204A, R505A). The complete amino acid sequence for HAB1* is provided in Supplementary Table 4.

### X-ray crystallography and structure determination

PYR1*^MANDI^ and HAB1* were expressed in *E. coli* and purified as described previously^25^. Purified protein was stored at −80 °C in a buffer containing 20 mM HEPES (pH 7.6), 50 mM sodium chloride, 10 mM DTT and 30% glycerol. Purified PYR1*^MANDI^ and HAB1* were mixed at a 1:1 molar ratio and exchanged into a buffer containing 20 mM HEPES (pH 7.6), 50 mM sodium chloride, 10 mM dithiothreitol and 5 mM magnesium chloride, mixed with a five-fold molar excess of mandi and concentrated to 15 mg ml^−1^. Crystallization of the PYR1*^MANDI^:mandipropamid:HAB1* ternary complex was conducted by sitting drop vapor diffusion at 19 °C. Drops were formed by mixing equal volumes of the ternary complex with a well solution containing 0.15 mM potassium bromide and 30% polyethylene glycol monomethyl ether 2,000. Crystals were flash frozen after passing through a well solution containing 20% glycerol. X-ray diffraction data for the ternary complex were collected from a single crystal at 100 K using the 21-ID-G beamline of the Advanced Photon Source at Argonne National Laboratories. Observed reflections were indexed, integrated and internally scaled using the HKL2000 software package (version 708)^57^. Molecular replacement was used to evaluate the initial phases of the PYR1*^MANDI^:mandipropamid:HAB1* ternary complex using the PYR1:mandipropamid:HAB1 ternary complex (Protein Data Bank (PDB) 4WVO) as the search model after removing mandipropamid and all waters. Phaser (version 2.6.0) solved the initial phases, and Phenix.AutoBuild (version 1.10.2155) automatically built the majority of residues for the complex^58^. The final model was completed through iterative rounds of manual model building in Coot (version 0.8.6)^59^ and refinement with Phenix.refine (version 1.15.2_3472)^58^ using individual atomic displacement and translation, libation, screw (TLS) parameters. Geometry of the final structure was validated using Molprobity^59–61^. Data collection and refinement statistics for the final model are listed in Supplementary Table 5, and the coordinates are deposited in the PDB (8EY0).

### PYR1*^MANDI^-SSM library and organophosphate screens

A saturating library harboring all possible single-site mutations (SSMs) of PYR1*^MANDI^ was made using a pool of 191 NNK oligonucleotides (Integrated DNA Technologies) by nicking mutagenesis^62^. The PYR1*^MANDI^ SSM libraries were deep sequenced using an Illumina MiSeq in paired-end mode (2 × 250) and analyzed using PACT^63^. Sequencing of the PYR1*^MANDI^ SSM library revealed 100% of the expected mutations at the amino acid level (3,629/3,629 single-point mutants). The resulting PYR1*^MANDI^-SSM plasmid library was transformed into an *S. cerevisiae* MaV99 strain harboring pACT-HAB1*, subjected to negative selection to purge constitutive mutants and screened against a panel of 10 organophosphates at 100 µM, using methods described previously^23,64^. The primary low-affinity azinphos-ethyl sensors isolated from the PYR1*^MANDI^-NNK library screen were shuffled using NeXT recombination-based mutagenesis; the libraries produced were introduced into Mav99(pACT-HAB1) and subjected to negative selection on 0.1% 5-FOA; and cells were collected and then subjected to positive selections on SD-LTU plates supplemented with 1 µM azinphos-ethyl. The hits obtained were subjected to another round of NeXT mutagenesis and negative/positive selections using 250 nM azinphos-ethyl to yield the final PYR1*^AZIN^ sensor. The complete amino acid sequence for PYR1*^AZIN^ is provided in Supplementary Table 4.

### *S. cerevisiae* genetic circuits

PYR1* variants were used to drive gene expression in an inducible genetic circuit by fusing a zinc finger DNA-binding domain (Z4 (ref. ^47^)) to the N-terminus of PYR1* and the VP64 activation domain^65^ to the N-terminus of ΔN-HAB1* (N-terminal truncation of the first 196 residues). The SV40 nuclear localization signal was also fused to the N-terminus of PYR1*. A single 2-µ plasmid was used to express SV40-Z4DBD-PYR1*^MANDI/AZIN^ and VP64-HAB1*. PYR1^ABA/DIAZI^ circuits were similarly built and expressed but used the EP DNA-binding domain in place of the Z4 domain. The reporter genes expression cassette for eGFP and mCherry was integrated into the YPRCΔ15 or YORCΔ22 site on chromosomes 16 and 15, respectively. Sequences for the promoters and terminators used to create the PYR1*/HAB1* and PYR1/HAB1 expression cassettes were derived from ref. ^66^. Plasmid construction started from pSW004, which expresses a WT PYR1 receptor and ΔN-HAB1 as previously described in ref. ^23^. The PYR1 variant, PYR1^DAIZI^, was amplified using SW021/SW022 and assembled with pSW004 digested with NheI and SacII. pSW012, which expresses ΔN-HAB1*, was used to amplify this gene using primers SW091/SW092 and replaced HAB1 in pSW004 after digestion with Spe1/Eag1. PYR1*^MANDI^ and PYR1*^AZIN^ were amplified using SW038/SW022 and inserted into pSW012 after digestion with BamH1/NHE1. All constructs were assembled using NEBuilder HiFi Assembly (New England Biolabs (NEB)) The GFP expression cassette with Z4 DNA binding sites was amplified from plasmid pSW010 as previously described^23^. The EP DNA-binding site sequence was amplified from pSW021 with SW221/SW222 and inserted into pSW010 with digestion by Not1/EcoR1. RFP was amplified with SW523/SW524 and inserted into pSW021 with digestion by Not1/Xho1. The primers used to construct these vectors, as well as constructed yeast strains, are provided in Supplementary Table 6.

### *S. cerevisiae* reporter gene assays

Three single transformants were picked from selection plates and inoculated in 2 ml of SD-U containing 2% glucose in 14 ml of culture tubes. After 24 h of growth at 30 °C in a shaker incubator (200 r.p.m.), the optical density at 600 nm (OD_600_) of each sample was measured, and cultures were back diluted to OD_600_ = 0.2 in fresh SD-U; the total volume of diluted culture was 9 ml. Each 9 ml of culture was separated into eight unique cultures by transferring 1 ml into eight wells of a 96-deep-well plate. Each well was used to evaluate the effect of a specific ligand concentration. After all the cultures were transferred, 1 µl of stock ligand solution (each stock varying in concentration) was added to each well. The plate was sealed with an air-breathable polymer film and cultured with 1,000-r.p.m. shaking at 30 °C for 12 h. Shaker humidity was maintained at 90%. Cells were harvested by centrifugation at 5,000*g* for 10 min, and, after discarding the supernatant, the cells were suspended in 1 ml of PBS buffer and centrifuged at 5,000*g* for 10 min. The cells were washed with 1 ml of PBS buffer twice and resuspended in 1 ml of DI water for flow cytometry analysis. For flow cytometry analysis, 50 µl of resuspended cells were transferred to a 96-well plate with a flat bottom, adding DI water up to a final volume of 200 µl. The fluorescence intensity of cells within each sample was measured using a BD Accuri C6 flow cytometer equipped with autoloading from 96-well plates. The forward scatter, side scatter, eGFP fluorescence (excitation/emission 488/533 nm) and mCherry fluorescence (excitation/emission 488/630 nm) were recorded for a minimum of 10,000 events.

### Generation and analysis of 35S-GFP::PYR1^DIAZI^ lines

Diazinon-regulated ABA responses were engineered by constructing a 35S::PYR1^DIAZI^-t_nos_ cassette. The PYR1^DIAZI^ coding sequence was PCR amplified using pBD-PYR1^DIAZI^ as a template. PCR fragments were purified and cloned into a derivative of the plant transformation vector pEGAD^67^, forming GFP-PYR1 fusion driven by the 35S promoter. The resulting construct was sequenced and transformed into the *Agrobacterium tumefaciens* GV3101 strain and transformed into Arabidopsis using the floral dip method. T_1_ seeds were plated on BASTA selection media, and resistant transgenic seedlings were propagated. Single-insert T_1_ lines were identified by analyses of GFP segregation in T_2_ self-progeny; homozygous lines were identified by segregation analyses of T_3_ progeny. To establish the effects of PYR1^DIAZI^ activation on ABA signaling, we used thermography measurements that report indirectly on ABA signaling in guard cells via changes in leaf temperature due to ABA-induced reductions in transpiration. Three-week-old homozygous transgenic plants and WT Columbia controls grown at 16-h light/8-h dark cycles were treated with foliar atomized (sprayed) applications of an aqueous solution containing 0.02% Silwet-77 and either carrier solvent (0.05% DMSO) or 500 nM diazinon, with four replicate pots. Each 4-inch pot contained both a single WT and a 35S::PYR1^DIAZI^ transgenic plant. Thermography data were collected using a FLIR T62101 camera 24 h after treatment, and mean leaf temperatures were estimated by averaging at least 14 ~1-cm-diameter spots per plant in FLIR’s software suite. GraphPad Prism 9 was used to conduct unpaired Student’s *t*-tests on the thermography data (*n* = 4 replicates).

### PYR1*^AZIN^/HAB1*-driven gene expression in plants

Two GAL4-based azinphos-ethyl regulated genetic circuits were constructed: one that drives expression of the synthetic RUBY construct in response to azinphos-ethyl and a second construct with a GFP reporter. The RUBY gene encodes a multi-gene product transcriptional unit that encodes enzymes that produce the beet betalain pigment when expressed in plants^53^; both constructs were made in the pCAMBIA1300 plant transformation vector backbone modified to replace the selection cassette with a 35S::mCherry-nos reporter cassette. A base plasmid with four separate cassettes was assembled using a combination of conventional cloning and Gibson assembly using NEBuilder HiFi assembly (NEB). The two GAL4-based plasmids harbor the following four cassettes and differ only in the presence of GFP or RUBY: UBQ10p::VP64-HAB1*-t_nos_; UAS_GAL_35S_min_::GFP-t_nos_ or UAS_GAL_35S_min_::RUBY-t_nos,_; PEX4p::GAL4_DBD-_PYR1*^AZIN^-t_RBCS_; and 35S::mCherry-t_nos_ (arranged in that order on the T-DNA from RB to LB). A third E-protein-based PYR1*^AZIN^/HAB1* module driving a RUBY reporter was generated similarly, using conventional and Gibson cloning with the following architecture: UBQ10p::VP64-HAB1*-t_nos_; 7xETR8-35S_min_::RUBY-t_nos_; PEX4p::EP_DBD-_PYR1*^AZIN^-t_RBCS_; and 35S::mCherry-t_nos_. The vectors made were sequenced (Plasmidsaurus), and their sequences are provided in Supplementary File 1.

The GAL4-based plasmids were transformed into *A. tumefaciens* (GV3101) and used to transform Arabidopsis using the floral dip method^68^. T_1_ plants were selected by identifying RFP^+^ seedlings; seeds from multiple T_1_ plants were collected; and segregation ratios in the T_2_ seedlings were used to identify single-insert transgenic lines, which were propagated another generation to obtain two homozygous lines for both the RUBY and GFP reporter lines. Seedlings were grown in a growth chamber (Percival Scientific) programmed with 8-h dark/16-h light cycles at 24 °C. Treatment and mock foliar applications were done using a hand-held atomizer using azinphos-ethyl or carrier solvent (DMSO) diluted in water with 0.02% Silwet wetting agent. In the case of the E-protein-driven system, primary T_1_ plants were analyzed.

### Dual organophosphate-regulated circuits transgenic plants

The PYR1^DIAZI^ coding sequence (see ref. ^23^ for sequence details) was PCR amplified and cloned into the pEGAD vector forming a 35S::PYR1^DIAZI^ fusion (lacking GFP). The resulting construct pEGAD-35S::PYR1^DIAZI^ was sequence confirmed and transformed into *A. tumefaciens* (GV3101) and was used to transform a heterozygous T_2_ PYR1*^AZIN^/HAB1* GFP line by the floral dip method. Transformants harboring both transgenes were selected in the T_1_ seed using glufosinate resistance (present on the 35S::PYR1^DIAZI^ construct) and mCherry expression (present on the PYR1*^AZIN^ construct). Two dual homozygous lines (harboring independent transformation events) were identified by marker segregation analyses, as described above. A RUBY-based double circuit combination was obtained by crossing an independently generated 35S::PYR1^DIAZI^ T_1_ line to a PYR1*^AZIN^/HAB1*-RUBY line and identifying homozygous F2s plants by marker segregation analyses.

### qRT–PCR analyses

To interrogate dual circuit functioning in planta by qRT–PCR analyses, Arabidopsis seeds were germinated on ½ Murashige–Skoog (MS) liquid media (0.25% sucrose, pH 5.7) and transferred to fresh MS media containing mock control, diazinon (500 nM), azinphos-ethyl (100 nM) or both compounds together (500 nM diazinon, 100 nM azinphos-ethyl). Seedlings were grown in a growth chamber (Percival Scientific) programmed with 8-h dark/16-h light cycles at 24 °C. Seedlings were collected 24 h after treatment, and total RNA was extracted using the RNeasy Plant Mini Kit (Qiagen, 74904). cDNA was synthesized from 500 ng of the RNA using the QuantiTect Reverse Transcription Kit (Qiagen, 205311). Quantitative RT–PCR reactions were conducted using the Maxima SYBR Green/Fluorescein qPCR Master Mix (Thermo Fisher Scientific, K0242) in three technical replicates using a Bio-Rad Real-Time PCR Detection System. The relative expression level of the ABA-responsive gene RD29B (using primers RD29B qPCR5 and RD29B qPCR3) and GFP (using primers GFP qPCR5 and GFP qPCR3) was normalized against the reference gene AtPEX4 (using primers Pex4 qPCR3 and Pex4 qPCR5) for each sample. Expression relative to the mock control was calculated using the 2^−ΔΔCT^ method.

### Chemicals

The organophosphate and mandipropamid ligands used in our screening experiments were obtained as analytical standard-grade materials (PESTANAL) from Sigma-Aldrich and were used without further purification. The ligands used in our sensor screens and subsequent biological experiments, as well as their PubChem CIDs, are as follows: mandipropamid (11292824), diazinon (3017), azinphos-ethyl (17531), pirimiphos-methyl (34526), malathion (4004), chlorfenvinphos (5377791), disulfoton (3118), dimethoate (3082), parathion-methyl (4130), bromophos-methyl (16422) and monocrotophos (5371562).

### Reporting summary

Further information on research design is available in the Nature Portfolio Reporting Summary linked to this article.

## Online content

Any methods, additional references, Nature Portfolio reporting summaries, source data, extended data, supplementary information, acknowledgements, peer review information; details of author contributions and competing interests; and statements of data and code availability are available at 10.1038/s41589-023-01447-7.

### Supplementary information

Supplementary Information Supplementary Figs. 1 and 2, Tables 1–8 and References.

Reporting Summary

Supplementary Data Vector sequences for constructs used in this paper and source data for supplementary figures.

### Source data

Source Data Fig. 1 PP2C inhibition source data for Fig. 1c.

Source Data Fig. 2 PP2C inhibition source data for Fig. 2c.

Source Data Fig. 4 Normalized fluorescence source data for Fig. 4b,d.

Source Data Fig. 5 Leaf temperature/qRT–PCR source data for Fig. 5a,c.

Source Data Extended Data Fig. 1 PP2C inhibition source data.

Source Data Extended Data Fig. 5 Source western blot.

Source Data Extended Data Fig. 8 Percent germination source data for Extended Data Fig. 8a,b.

## Data Availability

All data discussed in this paper are provided as supplementary information. The X-ray crystallographic coordinates are available from the Protein Data Bank website using accession code 8EY0. All materials are available upon reasonable request and the completion of standard material transfer agreements with the University of California, Riverside. Source data are provided with this paper.
