# Supplementary material for: An orthogonalized PYR1-based CID module with reprogrammable ligand-binding specificity
Source: Nat Chem Biol. 2023 Oct 23;20(1):103–10. doi: 10.1038/s41589-023-01447-7 (PMC10746540; doi:10.1038/s41589-023-01447-7)
Supplement: Supplementary file 1 — Supplementary Figs. 1 and 2, Tables 1–8 and References. [file 41589_2023_1447_MOESM1_ESM.pdf]

nature chemical biology

Article https://doi.org/10.1038/s41589-023-01447-7

An orthogonalized PYR1-based CID module with reprogrammable ligand-binding specificity

In the format provided by the authors and unedited

Supplementary Materials

Table of Contents

1. Supplementary Figures

2. Supplementary Tables

3. References

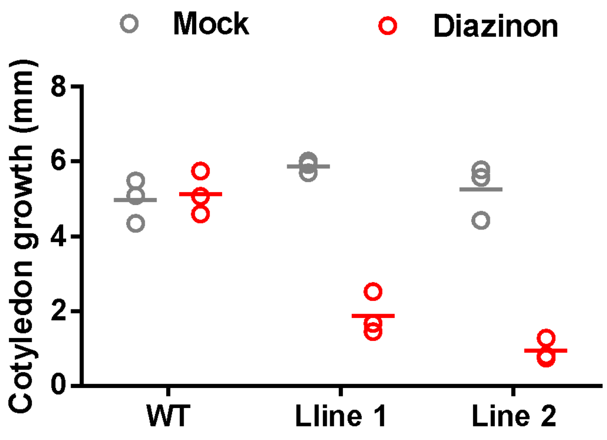

Supplementary Figure 1. Diazinon mediated growth responses in GFP PYR1^{DIAZI} transgenic lines. 6-day old seedlings from two independent transgenic lines (Line1, Line 2) were tested for ABA-like mediated growth responses on diazinon (500 nM) or mock. Wild-type (WT) seedlings were used as control. Each experiment consisted of 3 replicates with three plants each. Cotyledon's new growth was measured after seven days of treatment.

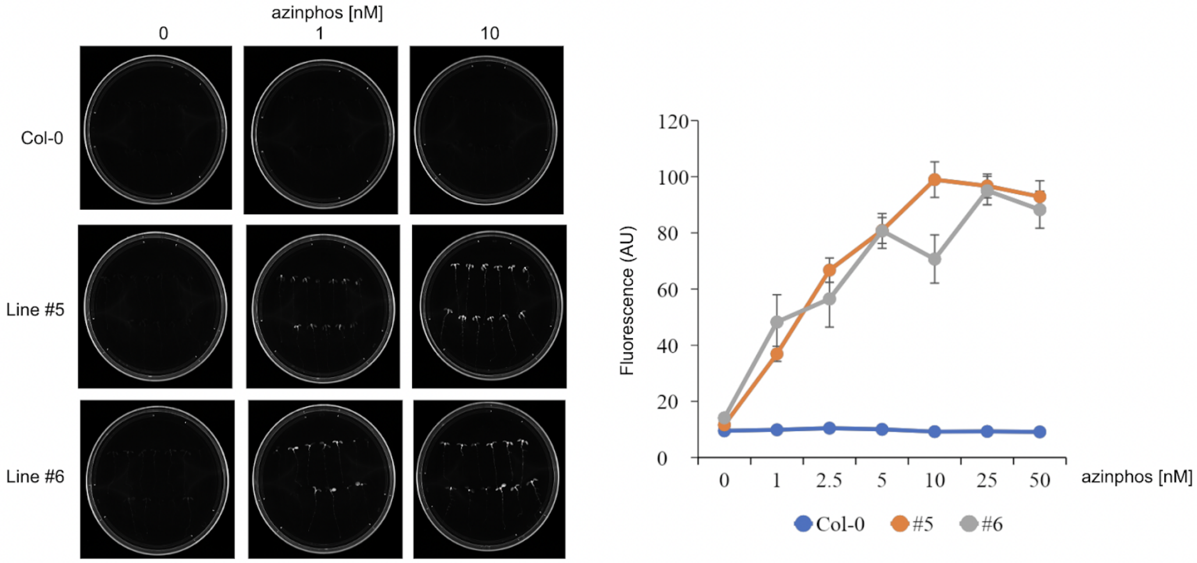

Supplementary Figure 2. A PYR1*^{AZIN}-based circuit drives eGFP gene expression in Arabidopsis in response to azinphos-ethyl. Shown are data from two single-insert homozygous transgenic lines (line 5 and line 6) with a GAL4-based PYR1*^{AZIN}/HAB1* circuit driving the expression of eGFP. Foliar applications of 0, 1, or 10 nM azinphos-ethyl were imaged 48 hours post-treatment (left); all solutions contain 0.1% DMSO (carrier solvent) and 0.02% Silwet (improves chemical uptake into inner tissues). Epifluorescence images were acquired using a BioRad GelDoc Imager. Twelve seedlings from two homozygous lines are shown on the left for mock, and two concentrations are analyzed. The right graph shows the fluorescence mean intensity of the seedlings (normalized to the seedling area) under various treatments, as analyzed using ImageJ.

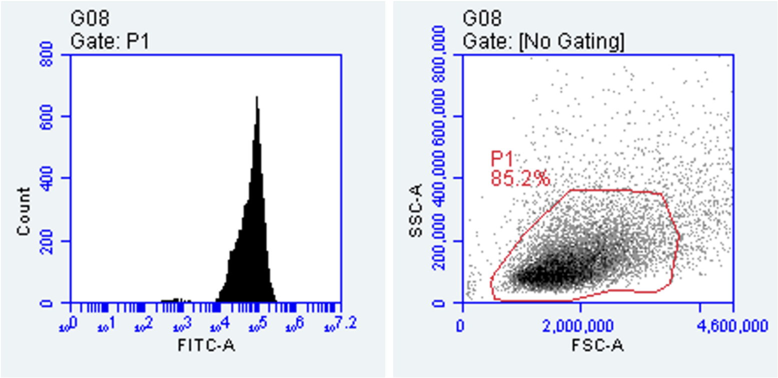

Supplementary Figure 3. Example of the gating strategy used to analyze flow cytometry data is shown in main Figure 4. This example shows the response of PYR1*MANDI to 1 μM mandipropamid.

Supplementary Table 1. Mutations in PYR1 that disrupt ABA-induced binding of PYR1 to HAB1. An indexed collection of pBD-PYR1 mutants was tested for interaction with pACT-HAB1 after growth on ABA (10 μM) using galactosidase assays; mutants that did not yield detectable b-gal staining were scored as non-functional and assigned an “x“ in the table.

	A	C	D	E	F	G	H	I	K	L	M	N	P	Q	R	S	T	V	W	Y
**F61**			x	x		x	x	x	x			x	x	x	x	x				
**I62**			x	x		x			x						x				x	x
**K63**													x							
**I84**										x										
**S85**	x	x	x	x	x	x	x	x	x	x	x	x	x	x	x			x	x	x
**G86**	x	x	x	x	x		x	x	x	x	x	x	x	x	x	x	x	x	x	x
**L87**	x	x	x	x		x	x		x			x		x	x	x	x		x	x
**P88**		x	x	x	x	x	x	x	x	x	x	x		x	x	x	x	x	x	x
**A89**		x	x	x	x		x	x	x	x	x	x	x	x	x	x	x	x		x
**H115**	x	x	x	x	x	x		x	x	x	x		x	x	x	x	x	x	x	x
**R116**	x	x	x	x	x	x	x	x					x			x	x	x	x	x
**L117**	x		x	x		x	x		x			x	x	x	x	x	x	x	x	x
**P148**		x	x	x	x	x	x	x	x	x	x	x		x	x	x	x	x	x	x
**G150**								x					x					x	x	
**N151**		x	x	x	x	x	x	x	x	x	x		x	x	x			x	x	x
**D155**		x			x	x		x	x	x	x		x		x			x	x	x
**T156**			x	x	x	x	x	x	x	x	x	x	x	x	x				x	x
**M158**													x							
**F159**			x	x		x	x		x			x	x	x	x	x			x	x
**T162**			x	x	x								x							
**K170**													x							

Supplementary Table 2. Mutations in HAB1 that disrupt binding to PYR1. An indexed collection of pACT-HAB1 mutants was tested for interaction with pBD-PYR1 after growth on ABA (10 μM) using galactosidase assays; mutants that did not yield detectable *β*-gal staining were scored as non-functional and assigned an “x“ in the table.

	A	C	D	E	F	G	H	I	K	L	M	N	P	Q	R	S	T	V	W	Y
**E201**							x		x											
**E203**	x	x	x		x	x	x	x	x	x	x	x	x		x	x	x	x	x	x
**H245**	x	x	x	x	x	x		x	x	x	x	x	x	x	x	x	x	x	x	x
**G246**	x	x	x	x	x		x	x	x	x	x	x	x	x	x	x	x	x	x	x
**G247**	x			x	x			x	x		x		x	x	x		x	x	x	x
**E323**										x										
**K381**																			x	
**I383**			x	x		x	x		x			x	x	x	x	x	x		x	x
**W385**	x	x	x	x	x	x	x	x	x	x	x	x	x	x	x	x	x	x		x
**R389**	x	x	x	x	x	x	x	x	x	x	x		x	x	x		x	x	x	x
**F391**			x	x									x							
**G392**	x	x	x	x	x		x	x	x	x	x	x	x	x	x		x	x	x	x
**V393**			x	x		x			x			x	x	x	x	x				
**Y404**			x	x		x			x			x	x	x	x	x	x			

Supplementary Table 3. Orthogonalizing mutation pairs defined using positive selections in *S. cerevisiae*.

PYR1	HAB1	Strength
WT	WT	++++
F61K	V393Q	++
S85P	E203D	+
S85P	E203T	+
S85P	E203W	+
T156P	I383G	+
T162D	V393K	++
T162D	V393R	+++

Supplementary Table 4. Sequences of engineered PYR1 and HAB1 variants.

<table>
<tr><th>Protein</th><th>Sequence</th></tr>
<tr><td></td><td>.........|.........|.........|.........|.........|</td></tr>
<tr><td>PYR1*^{MANDI}</td><td>MPSELTPEERSELKNSIAEFHTYQLDPGSCSSLHAQRIHAPPELVWSIVR
RFDKPQTHRHFIKSCSVEQNFEMRVGCTRDIIVISGLPANTSTERLDILD
DERRVTGASIIGGEHRLTNYKGVTTVHRFEKENRIWTVVLESYVVDMPEG
NSEDDTRIVVDDVVKLNLQKLATVAEAMARNSGDGSGSQVT</td></tr>
<tr><td>PYR1*^{AZIN}</td><td>MPSELTPEERSELKESIAEFHTYQLDPGSCSSLHAQRIHAPPELVWSIVR
RFDKPQTHRHFIKSCSVEQNFEMRVGCTRDIIWISGLPANTSTERLDILD
DERRVTGASYIGGEHSLTNYKGVTTVHRFEKENRIWTVVLESYVVDMPEG
NSEDDTRIVVDDVYKLNLQKLATVAEAMARNSGDGSGSQYT</td></tr>
<tr><td>PYR1^{DIAZI}</td><td>MPSELTPGERSELKNSIAEFHTYQLDPGSCSSLHAQRIHAPPELVWSIVR
RFDKPQTYKHFIKSCSVEQNFEMRVGCTRDYIVISGMPANTSTERLDILD
DERRVTGYSIIGGEHRLTNYKSVTTVHRFEKENRIWTVVLESYVVDMPEG
NSEDDTRVGVDTVVKLNLQKLATVAEAMARNSGDGSGSQVT</td></tr>
<tr><td>HAB1*</td><td>MEEMTPAVAMTLSLAANTMCESSPVEITQLKNVTDAADLLSDSENQSFCN
GGTECTMEDVSELEEVGEQDLLKTLSDTRSGSSNVFDEDDVLSVVEDNSA
VISEGLLVVDAGSELSLSNTAMEIDNGRVLATAIIVGESSIEQVPTAEVL
IAGVNQDTNTSEVVIRLPDENSNHLVKGRSVYELDCIPLWGTVSIQGNAS
EMEAAFAVSPHFLKLPIKMLMGDHEGMSPSLTHLTGHFFGVYDGHGGHKV
ADYCRDRLHFALAEEIERIKDELCKRNTGEGRQVQWDKVFTSCFLTVDGE
IEGKIGRAVVGSSDKVLEAVADETVGSTAVVALVCSSHIVVSNCGDSRAV
LFRGKEAMPLSVDHKPDREDEYARIENAGGKVIQWQGARVFGRLAMSRSI
GDRYLKPYVIPEPEVTFMPRSREDECLILASDGLWDVMNNQEVCEIARRR
ILMWHKKNGAPPLAERGKGIDPACQAAADYLSMLALQKGSKDNISIIVID
LKAQAKFKTRT</td></tr>
</table>

Supplementary Table 5. Data collection and refinement statistics

PYR1*^{MANDI}:mandipropamid:HAB1*	
**Data collection**	
Space group	P 2_1 2_1 2_1
**Cell dimensions**	
a, *b*, *c* (Å)	63.41, 92.21, 96.77
α, *β*, *γ* (°)	90, 90, 90
Resolution (Å)	50-2.40 (2.44-2.40)
R_{merge}	0.073 (0.847)
R_{meas}	0.078 (0.909)
R_{pim}	0.028 (0.326)
I / σI	26.4 (2.2)
Completeness (%)	99.9 (100)
Redundancy	7.5 (7.6)
**Refinement**	
Resolution (Å)	30.13-2.40 (2.46-2.40)
No. reflections	21,877
R_{cryst} / R_{free}	0.172/0.234
No. atoms	3827
Protein	3680
Ligand/ion	41
Water	106
B-factors	
Protein	63.8
Ligand/ion	62.9
Water	50.3
**R.m.s. deviations**	
Bond lengths (Å)	0.007
Bond angles (°)	0.843
**Ramachandran Stats**	
Favored	96.9%
Allowed	3.1%

Values in parentheses are for the highest resolution shell.

A single crystal was used for each structure determination.

Supplementary Table 6. Primers used in this work.

Primer	Sequence (5' -> 3')
sw021	GTTTTAATTACAAACCTAGGATGCCTTCGGAGTTAACACCAG
sw022	CCTCATCAAGATTGCTTTATGCTAGCTCACGTCACCTGAGAACCACTT
sw038	GTGTTAACTCCGAAGGCATCATCAAAGTTAACATGTCCAGGTCGAAA
sw091	GTATAAGAATCATTCAAAGGCGCGCATGACTCCCGCAGTTGCAATGA
sw092	ACATAACTAATTACATGAGGCTAGCTCAGGTTCTGGTCTTGAACTTTGC
sw221	AGGGAACAAAAGCTGGAGCTCGCCAGGCGCCTTTATATCATAT
sw222	AAGTAGTGACAAGTGTTGGCCATGGAACAGGTAGTTTTCCAGTAG
sw523	TTGGGTATAAAAGACCACGC GGCCGCATGGCGCGTAAGGTCGATCTCA
sw524	GTCCTCGGTGTTGTCCATGCTTAGCGTTGGTGGTGGGCGG
Hab1 cd5 EcoRI	AAAAAAGAATTCATGGAGGAGATGACTCCCGCAG
Hab1 cd3 XhoI	AAAAAACTCGAGTCAGGTTCTGGTCTTGAACT
Pex4 qPCR3	GGCGAGGCGTGTATACATTT
Pex4 qPCR5	CAGTCCTCTTAACTGCGACTCA
RD29B qPCR5	TGGTGGGGAAAGTTAAAGGA
RD29B qPCR3	GGAATCCGAAAACCCCATAGTCC
GFP qPCR5	CTATATCATGGCCGACAAGCAG
GFP qPCR3	GGTGTTCTGCTGGTAGTGGTCG
R199#	CATTCAAGGGAATNNKTCTGAGATGGAGG
S200#	CAAGGGAATAGANNKGAGATGGAGGATG
E201#	CAAGGGAATAGATCTNNKATGGAGGATGCTTTTG
E203#	GAATAGATCTGAGATGNNKGATGCTTTTGCCGTG
D243#	CATTTTTTCGGTGTTTATNNKGGTCATGGAGGCCA
G244#	TTCGGTGTTTATGATNNKCATGGAGGCCATAAG
H245#	GTGTTTATGATGGTNNKGGAGGCCATAAG
G246#	GTTTATGATGGTCATNNKGGCCATAAGGTTGCTG
G247#	GATGGTCATGGANNKCATAAGGTTGCTG
S322#	CTTGAGGCTGTTGCGNNKGAGACCGTAGGATC
E323#	GGCTGTTGCGTCTNNKACCGTAGGATCAAC
T324#	GTTGCGTCTGAGNNKGTAGGATCAACTG
D346#	GTTTCTAACTGCGGTNNKTCGAGGGCGGTTTTATTC
K365#	CTTGTCAGTTGATCACNNKCCAGATAGAGAGGA
K381#	GAAAATGCTGGAGGCNNKGTTATACAATGGCAAG
I383#	GGAGGCAAAGTTNNKCAATGGCAAGGCG
Q384#	GGCAAAGTTATANNKTGGCAAGGCGCAC
W385#	CAAAGTTATACAANNKCAAGGCGCACGTG
Q386#	CAAAGTTATACAATGGNNKGGCGCACGTGTT
R389#	CAATGGCAAGGCGCANNKGTTTTTGGTGTTCT
F391#	AGGCGCACGTGTTNNKGGTGTTCTCGCCA
G392#	CGCACGTGTTTTTNNKGTTCTCGCCATGTC
V393#	CACGTGTTTTTGGTNNKCTCGCCATGTCTAG
Y404#	CATCGGTGACAGANNKCTGAAGCCATATGTG
S431#	GTCTCATACTAGCCNNKGACGGTCTTTGGGATG
G433#	ATACTAGCCAGTGACNNKCTTTGGGATGTAATG
D436#	AGTGACGGTCTTTGGNNKGTAATGAACAACCAAG
D492#	CAAAAAGGAAGTAAANNKAACATCTCCATCATTG
N493#	CAAAAAGGAAGTAAAGACNNKATCTCCATCATTGTG
Pyr1 M158#_F159#_A160#	TCGGAGGATGATACTCGTNNKNNKNNKGATACGGTTGTGAAGCTT
RUBY-F	GAGAGAACACGGGGACTCTAGCGCTACCGGTATGGATCATGCGACCCTCGCCATGATC
RUBY-R	GATCGGGGAAATTCGCCTCGACCTAGGATCCTCACTATCACTGGAGGCTTGGCTCAAG
RUBY-cloning-R	TCTTGGCGAGGTTGGCGAAGGACCGAT
JDQ186-pRBSCt-HiFi-PYR*-F	CGGAAGTGGTTCTCAGGTGACGTGAAGATCCTAGAGAG
JDQ187-pBD-HiFi-PYR*-R	CTTCTGGTGTTAACTCCGAAGGCATGAATTGCG
JDQ188-PYR*-F	ATGCCTTCGGAGTTAACACCAGAAGAACGA
JDQ189-PYR*-R	TCACGTCACCTGAGAACCACTTCCGTCACCGG
JDQ190-mCherry-F	ATGTTGAGCAAGGGCGAGGAGGATA
JDQ192-mCherry-HiFi-2A-R	TATCCTCCTCGCCCTTGCTCAACATTGGTCCAGGATTCTCTTCGACGTCCCCC
JDQ203-pCAMBIAb-VP64-HiFi-F	AGCAAAGTTCAAGACCAGAACCTGA
JDQ204-pCAMBIAb-VP64-HiFi-R	CTCAGGTAAATCACGACCAGAACCA
JDQ207-pCAMBIAb-BD-HiFi-F	CGGAAGTGGTTCTCAGGTGACGTGA
JDQ208-pCAMBIAb-BD-HiFi-R	GAATTGCGGCGATACAGTCAACTGT
JDQ211-pCAMBIA3300b-BsaI-HiFi-F	CTCAGAAGACCAAAGGGCTATTGAG
JDQ212-pCAMBIA3300b-BsaI-HiFi-R	AAGCTTGGCACTGGCCGTCGTTTTA
JDQ213-Exp-cassette-EcoRI-HiFi-F	CCCCGGGTACCGAGCTCGGTCTCAGGACGAGTCAGTAATAAACGGCGT
JDQ214-Exp-cassette-EcoRI-HiFi-R	ATTACCCTTTGTTGAAAAGTCTCAGAATTGCGCAAGTCATAAAATGCAT
JDQ215-PYR-HiFi-F	CAATTCATGCCTTCGGAGTTAACA
JDQ216-tNOS-HiHi-R	CCGATCTAGTAACATAGATGACAC
JDQ217-35sm-HiHi-F	AGGCAAGACCCTTCCTCTATA
JDQ218-pPEX4-HiFi-R	ACCGGGGAGACCCTCTAATTTCCCAA
JDQ219-pPEX4-HiFi-EPDB-F	GGGAAATTAGAGGGTCTCCCCGGTATGCCCCGCCCCAAGCTCAAGTCCGATGAC
JDQ220-EPDB-HiFi-PYR1-R	GTGTTAACTCCGAAGGCATGAATTGGCTGTACGCGGACGCATGTGCCTGGAG
JDQ221-tNOS-HiFi-EPUAS-F	GGTGTCATCTATGTTACTAGATCGGCTCGGGAGGTTTAAACGATT
JDQ222-EPUAS-HiFi-35sm-R	CTTATATAGAGGAAGGGTCTTGCCTCTGCTTATATAGGGCTAGCATCC

Supplementary Table 7. Plasmids used in this work.

Plasmid name	Description	Reference
pSW024	p415GPD, HDup1-EPBS(4)-CYC1core-RFP-CYC1t-HDdown1	This Study
pSW023	p415GPD, HDup2-Z4(4)-CYC1core-RFP-CYC1t-HDdown2	This Study
pSW022	p415GPD, HDup2-EPBS(4)-CYC1core-RFP-CYC1t-HDdown2	This Study
pSW021	p415GPD, HDup1-EPBS(4)-CYC1core-GFP-CYC1t-HDdown1	This Study
pSW015	pRS426, TEF1p-SV40-Z4DBD-PYR1*AZIN-CYC1t, PGK1p-VP64-HAB1*-PGK1t	This Study
pSW014	pRS426, TEF1p-SV40-Z4DBD-PYR1*-CYC1t, PGK1p-VP64-HAB1*-PGK1t	This Study
pSW013	pRS426, TEF1p-SV40-Z4DBD-PYR1-CYC1t, PGK1p-VP64-HAB1*-PGK1t	This Study
pSW012	pRS426, TEF1p-SV40-Z4DBD-PYR1DAIZI-CYC1t, PGK1p-VP64-HAB1-PGK1t	This Study
pSW010	p415GPD, HDup1-Z4(4)-CYC1core-GFP-CYC1t-HDdown1	This Study
pSW004	pRS426, TEF1p-SV40-Z4DBD-PYR1-CYC1t, PGK1p-VP64-HAB1-PGK1t	Ref. 2
pIW156	p415GPD	Ref. 3
pACT-HAB1	Activation domain-HAB1 fusion isolated from a Y2H screen [1]	Ref. 1
pACT-HAB1*	pACT-HAB1* (R199A, S322D, V393R, D204A, R505A)	This study
pBD-PYR1		Ref. 1
pBD-PYR1-DIAZI		Ref. 2
pBD-PYR1*-MANDI		This Study
pBD-PYR1*-AZIN		This Study
pET28-PYR1 T162D	6xHis-PYR1 T162D	This Study
pET28-PYR1*	6xHis-PYR1 Y58H-K59R-V81I-F108A-S122G-M158I-F159V-A160V-T162D	This Study
pGex 4T-HAB1 V393R	GST-HAB1 V393R	This Study
pGex 4T-HAB1*	GST-HAB1 R199A-D204A-S322D-V393R-R505A	This Study
pEGAD-PYR1-DIAZI	35S::PYR1-DIAZI; 35S::BAR	This study
pEGAD-GFP-PYR1-DIAZI	35S::GFP-PYR1-DIAZI; 35S::BAR	This study
pEGAD-PYR1-MANDI	35S::PYR1-MANDI; 35S::BAR	Ref. 4
pEGAD-GFP-PYR1*-MANDI	35S::GFP-PYR1-MANDI*; 35S::BAR	This Study
pEGAD-GFP-HAB1*	35S::GFP-HAB1*; 35S::BAR	This Study
pCAMBIA1300mCherry-Gal4BD-PYR1*AZIN/VP64-HAB1*-Gal4UAS35Smin-RUBY	UBQ10p::VP64-Hab1*-NOSt/UAS-35S(m)p::RUBY-NOSt/PEX4p::BD-Pyr1*AZIN-rbcSt	This Study
pCAMBIA1300mCherry-Gal4BD-PYR1*AZIN/VP64-HAB1*-Gal4UAS35Smin-GFP	UBQ10p::VP64-Hab1*-NOSt/UAS-35S(m)p::GFP-NOSt/PEX4p::BD-Pyr1*AZIN-rbcSt	This Study
pCAMBIA1300mCherry-EPBD-PYR1*AZIN/VP64-HAB1*-EPUAS35Smin-RUBY	UBQ10p::VP64-Hab1*-NOSt/7x_etr8-35S(m)p::RUBY-NOSt/PEX4p::E4-BD-Pyr1*AZIN-rbcSt	This Study

Supplementary Table 8. Strains used in this work. Species names and strain genotypes are indicated with italic type.

Strain	Description	Reference
YS1165	*BY4742 YPRCΔ15::EPBS-CYC1core-eGFP-CYC1t; YPRCΔ22::Z4BS-CYC1core-mCherry-CYC1t*	This Study
YS1164	*BY4742 YPRCΔ15::Z4BS-CYC1core-eGFP-CYC1t; YPRCΔ22::EPBS-CYC1core-mCherry-CYC1t*	This Study
YS1163	*BY4742 YPRCΔ22::Z4BS-CYC1core-mCherry-CYC1t*	This Study
YS1162	*BY4742 YPRCΔ22::EPBS-CYC1core-eGFP-CYC1t*	This Study
YS1159	*BY4742 YPRCΔ15::EPBS-CYC1core-eGFP-CYC1t*	This Study
YS1155	*BY4742 YPRCΔ15::Z4BS-CYC1core-eGFP-CYC1t*	This Study
YS4	*S. cerevisiae BY4742 MATα his3Δ1 leu2Δ0 lys2Δ0 ura3Δ0*	GE Healthcare
MaV99	*S. cerevisiae MATa ura3-52 leu2-3,112 trpl-901 his3Δ200 ade2-101 gal4Δ gal80Δ canlRcyh2RLYS2::(GAL1::HIS3) GAL1::lacZ SPO13::10xGAL4site::URA3*	Ref. 5
Y190	*S. cerevisiae MAT**a**, gal4-542, gal80-538, his3, trp1-901, ade2-101, ura3-52, leu2-3, 112, URA3::GAL1-LacZ, Lys2::GAL1-HIS3cyh*	Ref. 6
pEGAD-GFP-PYR1-DIAZI-L1	*A. thaliana (Col-0); 35S::GFP-PYR1-DIAZI-BASTA*	This study
pEGAD-GFP-PYR1-DIAZI-L2	*A. thaliana (Col-0); 35S::GFP-PYR1-DIAZI-BASTA*	This study
PYR1-DIAZI + PYR1*AZIN-GFP-L1	*A. thaliana (Col-0); 35S::PYR1-DIAZI, 35S::BAR + GAL4-based-PYR1*AZIN, UAS-Gal-35Smin-GFP, 35S::mCherry (line 1)*	This study
PYR1-DIAZI + PYR1*AZIN-GFP-L2	*A. thaliana (Col-0); 35S::PYR1-DIAZI, 35S::BAR + GAL4-based-PYR1*AZIN, UAS-Gal-35Smin-GFP, 35S::mCherry (line 2)*	This study
PYR1-DIAZI + PYR1*AZIN-RUBY-L1	*A. thaliana (Col-0); 35S::PYR1-DIAZI, 35S::BAR + GAL4-based-PYR1*AZIN, UAS-Gal-35Smin-RUBY, 35S::mCherry (line 1)*	This study
pCAMBIA1300mCherry-Gal4BD-PYR1*AZIN/VP64-HAB1*-Gal4UAS35Smin-RUBY, #3	*A. thaliana (Col-0); GAL4-based-PYR1*AZIN, UAS-Gal-35Smin-RUBY , 35S::mCherry (line #3)*	This study
pCAMBIA1300mCherry-Gal4BD-PYR1*AZIN/VP64-HAB1*-Gal4UAS35Smin-RUBY, #21	*A. thaliana (Col-0); GAL4-based-PYR1*AZIN, UAS-Gal-35Smin-RUBY , 35S::mCherry (line #21)*	This study
pCAMBIA1300mCherry-Gal4BD-PYR1*AZIN/VP64-HAB1*-Gal4UAS35Smin-GFP, #5	*A. thaliana (Col-0); GAL4-based-PYR1*AZIN, UAS-Gal-35Smin-GFP , 35S::mCherry (line #5)*	This study
pCAMBIA1300mCherry-Gal4BD-PYR1*AZIN/VP64-HAB1*-Gal4UAS35Smin-GFP, #6	*A. thaliana (Col-0); GAL4-based-PYR1*AZIN, UAS-Gal-35Smin-GFP , 35S::mCherry (line #6)*	This study

References

1. Park, S.-Y. *et al.* Abscisic Acid Inhibits Type 2C Protein Phosphatases via the PYR/PYL Family of START Proteins. *Science* **324**, 1068–1071 (2009).
2. Beltrán, J. *et al.* Rapid biosensor development using plant hormone receptors as reprogrammable scaffolds. *Nat. Biotechnol.* (2022) doi:10.1038/s41587-022-01364-5.
3. Mumberg, D., Müller, R. & Funk, M. Yeast vectors for the controlled expression of heterologous proteins in different genetic backgrounds. *Gene* **156**, 119–122 (1995).
4. Park, S.-Y. *et al.* Agrochemical control of plant water use using engineered abscisic acid receptors. *Nature* **520**, 545–548 (2015).
5. Vidal, M., Brachmann, R. K., Fattaey, A., Harlow, E. & Boeke, J. D. Reverse two-hybrid and one-hybrid systems to detect dissociation of protein-protein and DNA-protein interactions. *Proc. Natl. Acad. Sci. U. S. A.* **93**, 10315–10320 (1996).
6. Harper, J. W., Adami, G. R., Wei, N., Keyomarsi, K. & Elledge, S. J. The p21 Cdk-interacting protein Cip1 is a potent inhibitor of G1 cyclin-dependent kinases. *Cell* **75**, 805–816 (1993).
