# Supplementary material for: An orthogonalized PYR1-based CID module with reprogrammable ligand-binding specificity
Source: Nat Chem Biol. 2023 Oct 23;20(1):103–10. doi: 10.1038/s41589-023-01447-7 (PMC10746540; doi:10.1038/s41589-023-01447-7)
Supplement: Supplementary file 2 — Reporting Summary [file 41589_2023_1447_MOESM2_ESM.pdf]

nature portfolio

Corresponding author(s): Sean Cutler and Ian Wheeldon

Last updated by author(s): Jul 13, 2023

Reporting Summary

Nature Portfolio wishes to improve the reproducibility of the work that we publish. This form provides structure for consistency and transparency in reporting. For further information on Nature Portfolio policies, see our Editorial Policies and the Editorial Policy Checklist.

Statistics

For all statistical analyses, confirm that the following items are present in the figure legend, table legend, main text, or Methods section.

n/a	Confirmed	
☐	☒	The exact sample size (*n*) for each experimental group/condition, given as a discrete number and unit of measurement
☐	☒	A statement on whether measurements were taken from distinct samples or whether the same sample was measured repeatedly
☐	☒	The statistical test(s) used AND whether they are one- or two-sided<br>*Only common tests should be described solely by name; describe more complex techniques in the Methods section.*
☒	☐	A description of all covariates tested
☐	☒	A description of any assumptions or corrections, such as tests of normality and adjustment for multiple comparisons
☐	☒	A full description of the statistical parameters including central tendency (e.g. means) or other basic estimates (e.g. regression coefficient) AND variation (e.g. standard deviation) or associated estimates of uncertainty (e.g. confidence intervals)
☐	☒	For null hypothesis testing, the test statistic (e.g. *F*, *t*, *r*) with confidence intervals, effect sizes, degrees of freedom and *P* value noted<br>*Give P values as exact values whenever suitable.*
☒	☐	For Bayesian analysis, information on the choice of priors and Markov chain Monte Carlo settings
☒	☐	For hierarchical and complex designs, identification of the appropriate level for tests and full reporting of outcomes
☒	☐	Estimates of effect sizes (e.g. Cohen's *d*, Pearson's *r*), indicating how they were calculated

Our web collection on statistics for biologists contains articles on many of the points above.

Software and code

Policy information about availability of computer code

Data collection	FLIR thermal suite camera software was used to acquire leaf temperatures. Tecan Magellan software was used to acquire microplate reader data. BD Accuri C6+ software was used to acquire flow cytometry data. Phaser (v. 2.6.0), Phenix.AutoBuild (v. 1.10.2155), Coot (v. 0.8.6), Phenix.refine (v. 1.15.2_3472) and Molprobity were used in x-ray crystal data acquisition and refinement.
Data analysis	Prism Graphpad (v. 9) was used to analyze data, generate graphs, and conduct statistical analyses.

For manuscripts utilizing custom algorithms or software that are central to the research but not yet described in published literature, software must be made available to editors and reviewers. We strongly encourage code deposition in a community repository (e.g. GitHub). See the Nature Portfolio guidelines for submitting code & software for further information.

Data

Policy information about availability of data

All manuscripts must include a data availability statement. This statement should provide the following information, where applicable:

- Accession codes, unique identifiers, or web links for publicly available datasets
- A description of any restrictions on data availability
- For clinical datasets or third party data, please ensure that the statement adheres to our policy

All data discussed in this manuscript is provided as supplementary information. The x-ray crystallographic coordinates are available from the PBD website using accession code 8EY0. Plasmid and mutant receptor sequences are provided as supplementary files.

Human research participants

Policy information about studies involving human research participants and Sex and Gender in Research.

Reporting on sex and gender	n/a
Population characteristics	n/a
Recruitment	n/a
Ethics oversight	n/a

Note that full information on the approval of the study protocol must also be provided in the manuscript.

Field-specific reporting

Please select the one below that is the best fit for your research. If you are not sure, read the appropriate sections before making your selection.

☒ Life sciences ☐ Behavioural & social sciences ☐ Ecological, evolutionary & environmental sciences

For a reference copy of the document with all sections, see nature.com/documents/nr-reporting-summary-flat.pdf

Life sciences study design

All studies must disclose on these points even when the disclosure is negative.

Sample size	No sample size calculations were performed. The sample size (n) of each experiment is provided in the corresponding figure captions or methods section in the main manuscript and supplementary information files. Sample sizes were chosen to support meaningful conclusions. For yeast-based fluorescence studies sample size as 3. For plant experiments the sample size greater to or equal to 3.
Data exclusions	No data were excluded from our analyses.
Replication	Based on prior experience with the yeast-based fluorescence assays used in this study, we elected to test at least three biological replicates. A biological replicate is a unique clone from a single DNA transformation. Based on prior experience with leaf temperature assays, we used biological quadruplicates. Each biological replicate contains two plants in a single pot; pots were randomized. All replicates were successful.
Randomization	All reported yeast experiments were conducted with clonal populations. This work does not involve participant groups; therefore, randomization was not relevant to the study. For plant experiments a random number generator was used to select a number corresponding to a pot location within the growth chamber. This was done to control for potential positional effects on leaf temperature.
Blinding	The work does not involve participant groups, therefore, blinding was not relevant to the study

Reporting for specific materials, systems and methods

We require information from authors about some types of materials, experimental systems and methods used in many studies. Here, indicate whether each material, system or method listed is relevant to your study. If you are not sure if a list item applies to your research, read the appropriate section before selecting a response.

Materials & experimental systems

n/a	Involved in the study	
☐	☒	Antibodies
☒	☐	Eukaryotic cell lines
☒	☐	Palaeontology and archaeology
☒	☐	Animals and other organisms
☒	☐	Clinical data
☒	☐	Dual use research of concern

Methods

n/a	Involved in the study	
☒	☐	ChIP-seq
☐	☒	Flow cytometry
☒	☐	MRI-based neuroimaging

Antibodies

Antibodies used	*Describe all antibodies used in the study; as applicable, provide supplier name, catalog number, clone name, and lot number.*

Validation

Describe the validation of each primary antibody for the species and application, noting any validation statements on the manufacturer's website, relevant citations, antibody profiles in online databases, or data provided in the manuscript.

Flow Cytometry

Plots

Confirm that:

☒ The axis labels state the marker and fluorochrome used (e.g. CD4-FITC).

☒ The axis scales are clearly visible. Include numbers along axes only for bottom left plot of group (a 'group' is an analysis of identical markers).

☒ All plots are contour plots with outliers or pseudocolor plots.

☒ A numerical value for number of cells or percentage (with statistics) is provided.

Methodology

Sample preparation	The cells were washed with 1 mL PBS buffer twice and resuspended in 1 mL DI water for flow cytometry analysis. For analysis, 50 uL of resuspended cells were transferred to a 96-well plate with flat bottom, adding DI water up to a final volume of 200 uL.
Instrument	Samples were analyzed using BD Accuri C6+ flow cytometry
Software	Samples were analyzed using BD Accuri C6+ analysis software
Cell population abundance	At least 10,000 cells were analyzed in each experiment.
Gating strategy	Standard gating to remove irregular sized cells was used. See Supplementary Figure 3 for an example of the gating strategy.

☒ Tick this box to confirm that a figure exemplifying the gating strategy is provided in the Supplementary Information.
