# Supplementary figures and images for: An orthogonalized PYR1-based CID module with reprogrammable ligand-binding specificity
Source: Nat Chem Biol. 2023 Oct 23;20(1):103–10. doi: 10.1038/s41589-023-01447-7 (PMC10746540; doi:10.1038/s41589-023-01447-7)

Seedling

(kD)

130

95

72

55

43

34

26

17

WT P1 P\* M2c WT H\*  
-0X -1 -2 -3 -2 -1 -2 -3 -4

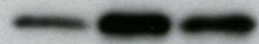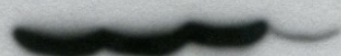

Supplement: Supplementary file 9 — Source western blot. [file 41589_2023_1447_MOESM9_ESM.pdf]
